# Effect of AcrySof versus other intraocular lens properties on the risk of Nd:YAG capsulotomy after cataract surgery: A systematic literature review and network meta-analysis

**DOI:** 10.1371/journal.pone.0220498

**Published:** 2019-08-19

**Authors:** Howard Thom, Frank Ender, Saisudha Samavedam, Caridad Perez Vivez, Subhajit Gupta, Mukesh Dhariwal, Jan de Haan, Derek O’Boyle

**Affiliations:** 1 Bristol Medical School, University of Bristol, Bristol, United Kingdom; 2 Alcon Management SA., Cointrin, Switzerland; 3 Novartis Healthcare Private Ltd., Hyderabad, India; 4 Alcon Laboratories Ireland Ltd., Cork, Ireland; 5 Alcon Vision LLC., Fort Worth, Texas, United States of America; 6 Alcon Nederland BV., Gorinchem, Netherlands; Keio University School of Medicine, JAPAN

## Abstract

**Objective:**

The purpose of this study was to evaluate the impact of different intraocular lens materials (IOL) and optic edge designs on the incidence of Nd:YAG laser capsulotomy.

**Methods:**

Randomized controlled trials (RCTs) reporting incidence of Nd:YAG capsulotomy in patients with monofocal IOLs were identified for systematic literature review (SLR) using Cochrane methodology. A network meta-analysis was conducted under a Bayesian framework. Mean hazard ratios (HRs), 95% credible intervals, and one-sided p-values were estimated for Nd:YAG capsulotomy incidence by comparing AcrySof IOLs with a group of non-AcrySof hydrophobic acrylic, hydrophilic acrylic, silicone, and PMMA IOLs. Sensitivity analysis was conducted comparing the risk of Nd:YAG capsulotomy between sharp- and round-edged designs of the above IOLs.

**Results:**

AcrySof IOLs had a lower risk of Nd:YAG capsulotomy compared to hydrophobic acrylic (HR: 2.68; 95% CrI: 1.41, 4.77; p < 0.01), hydrophilic acrylic (HR: 7.54; 95% CrI: 4.24, 14.06; p < 0.001), PMMA (HR: 3.64, 95% CrI: 1.87, 6.33; p < 0.001), and silicone (HR: 1.13; 95% CrI: 0.59, 1.91; p <0.1) IOLs. The risk for Nd:YAG was highest among sharp-edged IOLs for hydrophilic acrylic IOLs (HR: 9.32; 95% CrI: 4.32, 19.29; p < 0.01), followed by other hydrophobic acrylic (HR: 2.91; 95% CrI: 1.27, 5.88; p < 0.01), silicone (HR: 0.838; 95% CrI: 0.328, 1.74; p = 0.69), and PMMA (HR: 0.39; 95% CrI: 0.042, 1.49; p = 0.93) IOLs, compared to AcrySof. Acrysof IOLs had a lower risk of Nd:YAG compared to PMMA (HR: 3.25; 95% CrI: 1.21, 7.37; p < 0.01) and silicone, round edge IOLs (HR: 3.84; 95% CrI: 1.08, 10.64; p = 0.015).

**Conclusion:**

The risk of Nd:YAG capsulotomy is lower in eyes implanted with AcrySof IOLs compared to non-AcrySof hydrophobic or hydrophilic acrylic IOLs. Sharp-edged AcrySof, PMMA, and silicone IOLs are comparable in terms of reducing the risk of Nd:YAG laser capsulotomy.

## Introduction

Cataract surgery is one of the most frequently performed surgical procedures worldwide [1, 2]. Posterior capsule opacification (PCO) is the most common postoperative complication after cataract surgery with PCO rates of up to 43% being described within the first year after surgery [3]. PCO is characterized by abnormal proliferation of lens epithelial cells (LECs) on the posterior lens capsule blocking the visual axis, thus resulting in reduced visual acuity, impaired contrast sensitivity and glare disability. It is understood to be a multifactorial complication affected by several factors such as age, ocular comorbidities, surgical technique, and IOL material and design [4].

In terms of lens material, the earliest IOLs were made of polymethylmethacrylate (PMMA), which were first used in cataract surgery in the 1940s. However, with the introduction of phacoemulsification and the ability to extract the cataract through smaller incisions, foldable materials such as silicone, hydrophobic acrylic and hydrophilic acrylic were developed [5], among which, hydrophobic acrylic are predominant in use in the western world [6]. Concerning IOL design, the concept of a sharp optic edge has been shown to be an effective method to reduce PCO. As a result, the use of round-edge IOLs in current clinical practice has diminished significantly [5].

The role that IOL material plays in inhibiting PCO development has been evaluated in several clinical studies with varied results, although more favorable outcomes were observed for hydrophobic acrylic IOLs compared to other IOL materials due their superior bioactivity [4, 7–9]. AcrySof IOLs (Alcon Laboratories, Inc.) are made from a hydrophobic acrylic material with higher fibronectin bio-adhesion properties and a sharp optic edge [10]. Its bioactivity results in a sandwich pattern of adhesion between a single layer of LECs, the posterior capsule, and the IOL body allowing better binding compared to other materials and thus, inhibiting PCO development [10, 11]. In addition, IOLs manufactured with sharp optic edges have been shown to be more effective in preventing PCO by creating a mechanical barrier at the capsular bend compared to IOLs with round edges [12].The above findings were also confirmed in previous evidence synthesis studies that compared the PCO-preventing performance of different IOL materials and designs by using a meta-analysis methodology [7–9, 13].

At present, neodymium-doped yttrium aluminum garnet laser (Nd:YAG) capsulotomy is the only effective treatment for PCO and is commonly considered as a proxy measure for visually significant PCO [14, 15]. Although the procedure is generally considered safe, it is associated with a range of complications such as intraocular pressure elevation, intraocular lens (IOL) dislocation, retinal detachment, and cystoid macular edema that may require further medical and/or surgical treatment [16].

In addition to the clinical consequences, Nd:YAG capsulotomy places a significant financial burden on the limited resources of national healthcare systems. Indeed, payment for the Nd:YAG laser procedure in the Medicare program amounted to $187.5 million in 2010 (latest year available) [17]. Hence, the prevention of PCO and subsequent reduction in Nd:YAG laser capsulotomies is a major concern in current cataract surgical practice and also from the perspective of resource-constrained healthcare systems.

To our knowledge, none of the existing studies in peer-reviewed literature have included indirect evidence and conducted a comprehensive network meta-analysis (NMA). An NMA consists of statistical methods to combine and analyze data from all available randomized controlled trials (RCTs) to compare multiple treatment options [18–20]. When multiple comparisons between large numbers of treatments are performed it is important to account for both direct evidence from head-to-head RCTs and indirect evidence utilizing chains of RCTs sharing common comparators [19, 20]. Extensive guidelines are available for the conduct and reporting of NMA, which have been published by national health technology assessment agencies and international scientific organizations [21–23].

In Li et al. (2008), the authors pooled direct evidence from head-to-head RCTs to compare the efficacy of AcrySof IOLs with silicone and PMMA IOLs using a pairwise meta-analysis [7]. The study concluded that AcrySof IOLs were superior to round edged silicone and PMMA IOLs, demonstrating evidence of lower PCO and Nd:YAG capsulotomy rates [7]. We are extending this study using NMA to include non-Acrysof hydrophobic acrylic IOLs and hydrophilic acrylic IOLs as additional comparators, given their widespread use in current surgical practice [6] and the attention lens material has received in the published literature in terms of the role it plays in PCO formation and subsequent Nd:YAG capsulotomy rates [24–26]. The aim of the current SLR with NMA was to assess the impact of different IOL materials and optic edge designs on Nd:YAG capsulotomy rates.

## Methodology

### Literature search

The present study followed a standard, systematic literature review (SLR) methodology recommended by the Cochrane Collaboration and National Institute for Health and Care Excellence (NICE) in the UK [27, 28]. The SLR was conducted and reported in accordance with the Preferred Reporting Items for Systematic Reviews and Meta-Analyses (PRISMA) guidelines [29]. The literature search was conducted in the following electronic databases: Embase, Medline, Medline In-process, and Cochrane databases through Embase and Ovid platforms; from January 1996 through August 2017. A detailed search strategy using terms related to ‘cataract’, ‘intraocular lens’, ‘posterior capsular opacification’, and ‘Nd:YAG laser’ was developed to retrieve published literature that reported evidence matching our research objectives.

Eligible studies were selected based on the inclusion and exclusion criteria presented in Table 1. In a first pass, the titles and abstracts of the identified citations were screened by two independent reviewers and any discrepancies were reconciled by a third independent reviewer. Following first pass, full text reports of included citations were obtained for a more detailed evaluation. Each full-text report was reviewed in detail by two independent reviewers to decide its inclusion. Any discrepancy was reconciled by the third reviewer. At each step, reasons for excluding articles were noted. Further, the bibliography section of the included publications was cross-referenced to check if any relevant studies have been missed.

**Table 1:** Inclusion and exclusion criteria.

Category	Inclusion criteria	Exclusion criteria
**Patient population**	Age-related cataract patients	History of other ocular diseases, diabetes requiring medical control
**Intervention/comparator**	Cataract surgery with monofocal IOL implants	IOL implantation for non-age related cataract, and IOL implantation for presbyopia
**Outcome**	Incidence of Nd:YAG laser capsulotomy	**—**
**Study design**	Randomized controlled trials	Non-RCTs, publications in languages other than English, studies with follow-up of <6 months, studies with a sample size of <20 eyes, review articles, editorials, surveys, case-reports, and pilot studies

### Data extraction and quality assessment

Data from the included studies related to the full citation, abstract, objective, study location, methodology, follow-up duration, baseline characteristics such as age and gender, interventions, and incidence rates of Nd:YAG capsulotomy procedures at each reported follow-up time-point were extracted. With respect to intervention, IOL model properties were extracted as given in the publications and validated with the information available on manufacturer websites. RCTs with multiple publications were matched to avoid data duplication. Eligible RCTs meeting the inclusion criteria were critically appraised for quality as per the Cochrane’s ‘risk of bias assessment’ tool to assess the risk of bias for randomization, allocation concealment, blinding, incomplete outcomes data, and selective reporting. Further to this, publication bias was evaluated by constructing funnel plots for the studies included in each direct comparison for Nd:YAG capsulotomy S3 File. Data extraction was conducted in two steps: extraction of relevant data by the two reviewers; quality check by a third independent reviewer.

### Network meta-analysis

#### Overview

The incidence of Nd:YAG capsulotomy is reported at multiple time points for different IOL brands and models in the RCTs identified in the SLR. These RCTs formed an evidence network of both direct and indirect comparative evidence. NMA under the Bayesian framework was deemed the best way to synthesize this data and estimate relative treatment effects [21–23].

#### Constructing the evidence network

In order to construct networks for the NMA, IOL models were grouped based on the properties known to have an influence on PCO development and subsequent Nd:YAG incidence rates, namely, lens material and optic edge (sharp/round) design [7, 8], thus resulting in the following contrasts:

- Base-case analysis: AcrySof vs. other hydrophobic acrylic, hydrophilic acrylic (including those with modified surface, referred as hydrophilic acrylic hereon) silicone, and PMMA IOLs without optic edge classification.
- Sensitivity analysis: AcrySof vs. other hydrophobic acrylic, hydrophilic acrylic silicone, and PMMA IOLs with edge classification into round or sharp edged.

To develop a full evidence network for the two contrasts, it was essential that each comparator in a study belonged to different material type (base-case analysis) or was of a different edge property (sensitivity analysis). Studies comparing the same treatment groups (e.g., hydrophilic acrylic in both treatment arms) were excluded from the analysis as they do not provide evidence on the relative treatment effects that are estimated by NMA.

#### Statistical model

Given that the incidence of Nd:YAG capsulotomy is a dichotomous endpoint, a binomial likelihood model with a complementary log-log (cloglog) link incorporating an offset for study follow-up time was used [30]. Models using only the latest time point from each study and models incorporating multiple time-points per study were explored. The base case model assumed constant hazard ratios (HRs) for Nd:YAG over all time points but models with piecewise constant HRs over 0–1, 1–2, and >2 years were explored in sensitivity analysis [31]. NMA was conducted on evidence networks defined by both base-case analysis and sensitivity analysis and model choice was made on the basis of Deviance Information Criterion (DIC) and total residual deviance [32]. Using the selected model, means and 95% credible intervals (CrI) and one-sided p-values were estimated for the HRs. The Bayesian probability that hazard of Nd:YAG on AcrySof IOL was lower than comparator, the posterior probability that AcrySof IOL is superior, was also calculated; we name these Bayesian p-values as they provide a one-sided test of superiority.

We avoid the concept of “statistical significance” and instead adopt a strength of evidence approach in interpreting p-values and 95% CrI [33]. Inconsistency between the direct and indirect evidence in each evidence network was assessed in the selected model using an inconsistency model [34]. All analyses were conducted in RStudio version 0.99.878 running R 3.2.2. OpenBUGS version 3.2.3 was used to run the Bayesian NMA models. Further model details are provided in S1 File.

## Results

### Results of the literature search

**Fig 1:**
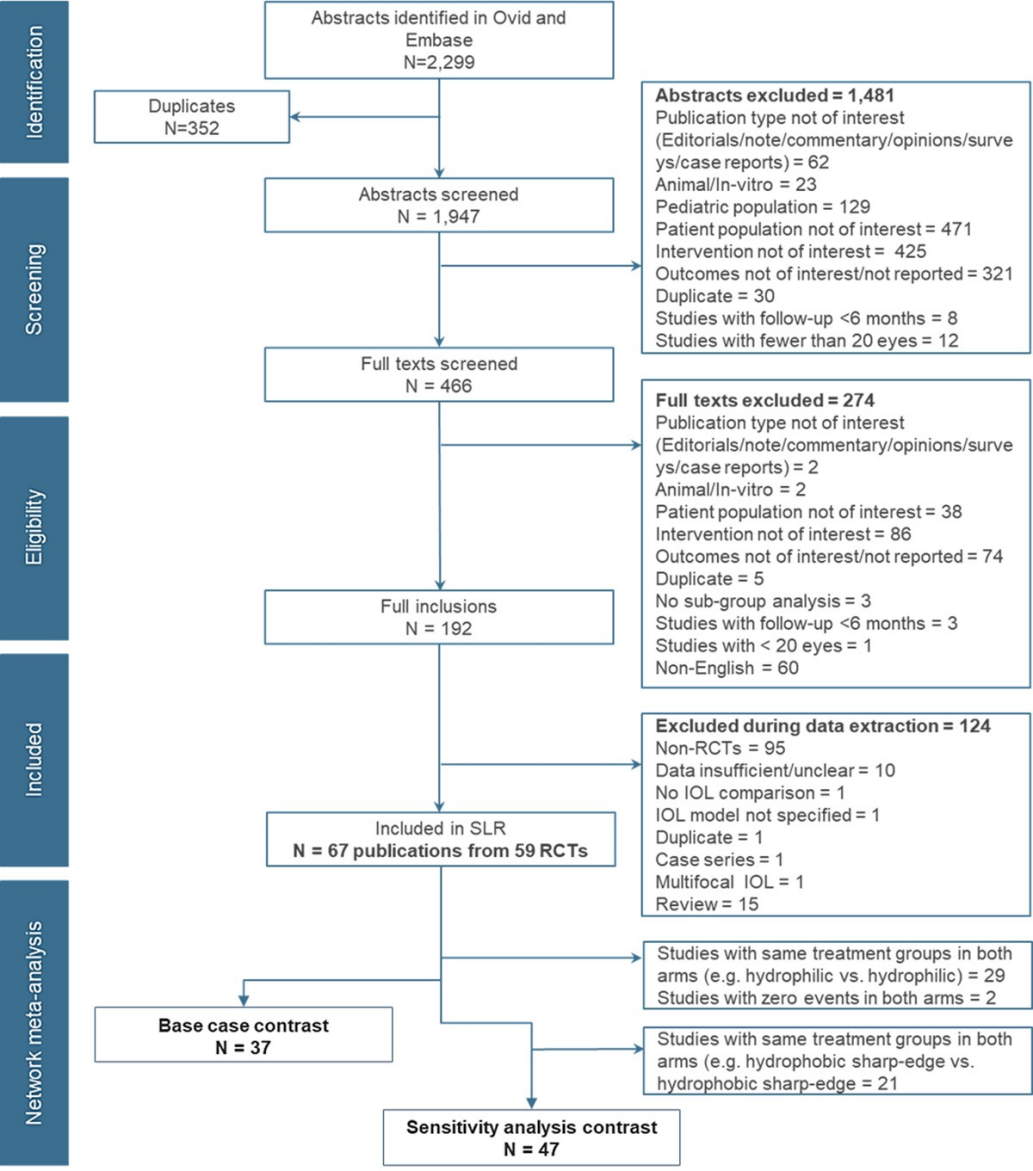
PRISMA flowchart for study selection.

A total of 2,299 citations were identified in the search from both Embase and Ovid platforms. After screening, 67 publications from 59 RCTs met the inclusion criteria. An overview of citation flow through various steps in the SLR process is presented in Fig 1.

### Quality assessment of included studies

**Fig 2:**
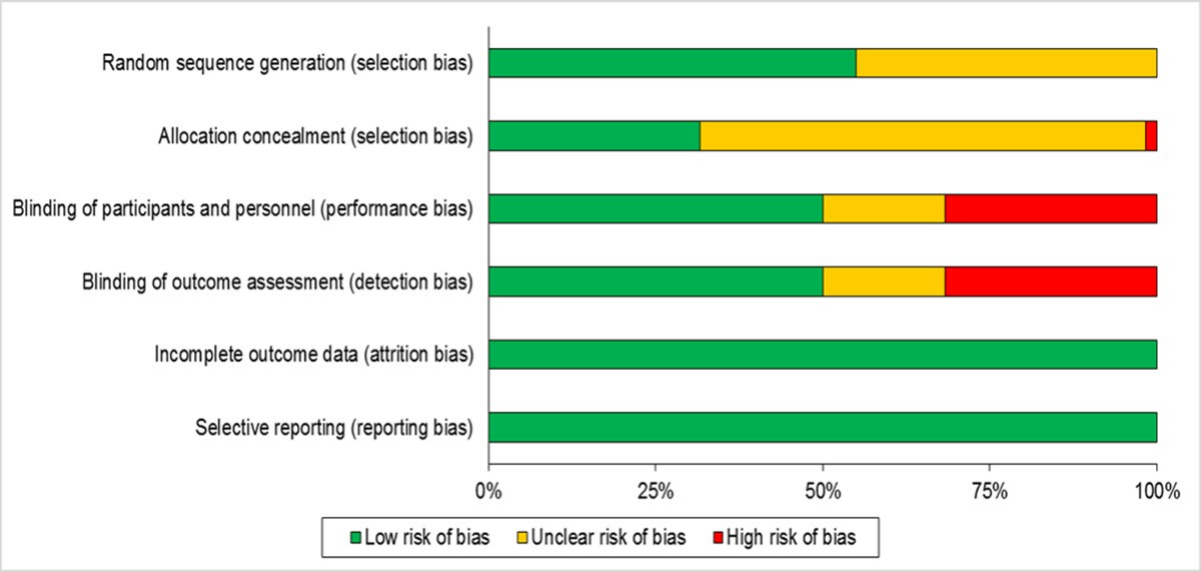
Risk of bias assessment of RCTs included in SLR.

Risk of bias assessment of the included RCTs is presented in Fig 2. Nearly half of the studies were at ‘low risk’ and other half were at ‘unclear risk’ for selection bias. Similarly, half of the studies were at ‘low risk’ for detection bias and performance bias. All the studies were at ‘low risk’ for attrition bias and reporting bias. All the studies were reviewed irrespective of the level of bias in order not to lose any evidence while constructing the evidence network. Individual contrast funnel plots are presented in S3 File. and indicate little evidence of publication bias.

### Descriptive overview

In summary, the total sample size in the included RCTs ranged from 40 eyes [35] to 2,778 eyes [36]. The follow-up duration ranged from 6 months [37] to 12 years [38]. The mean (±SD) age of the patients recruited in the studies ranged from 52.83 (±6.41) to 78 (±4) years. The AcrySof IOL was the most commonly used intervention compared to other IOLs of various material and designs, reported in 38 RCTs. Among the remaining 22 RCTs, the interventions and comparators were other hydrophobic acrylic IOLs, hydrophilic, silicone, and PMMA IOLs. Baseline details of each RCT and IOL properties are presented in S2 File.

### Results of the NMA

**Fig 3:**
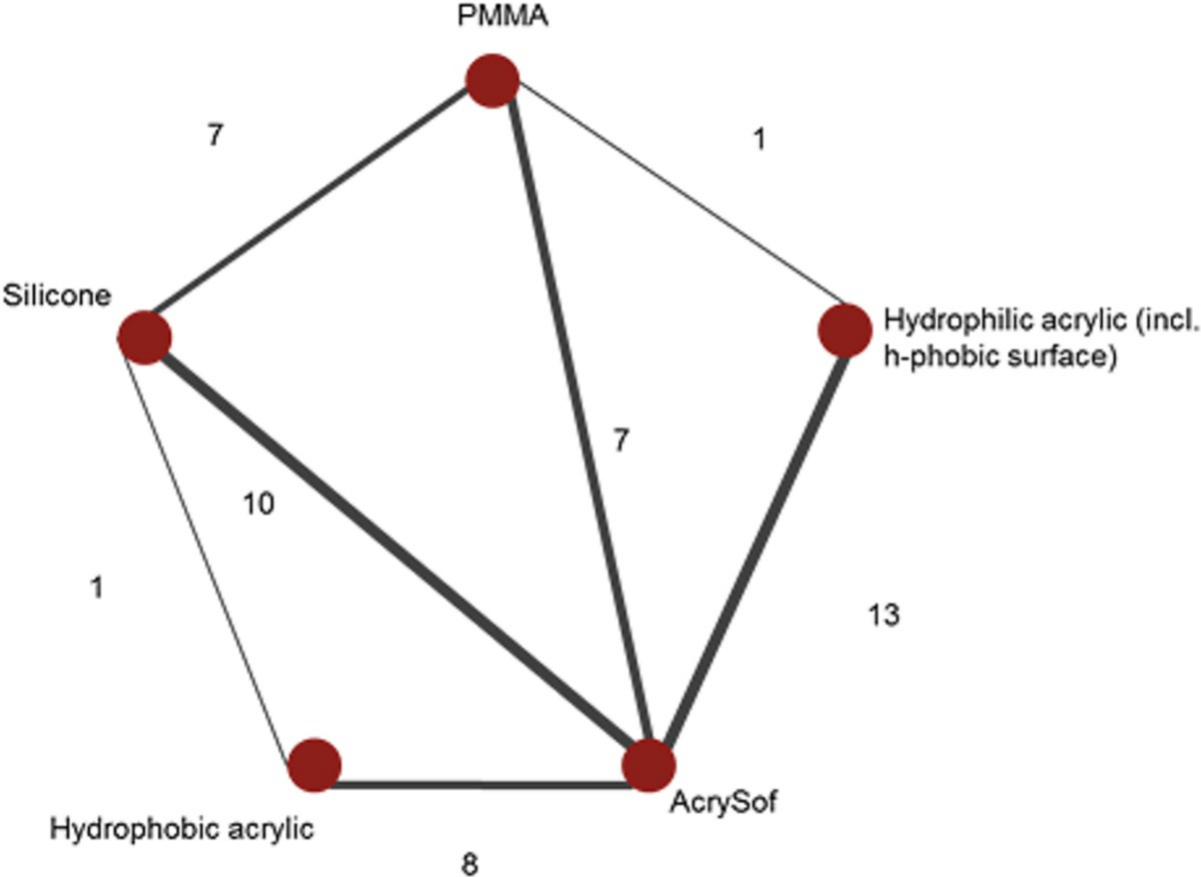
Evidence network for Base-case analysis: AcrySof vs. other hydrophobic acrylic, hydrophilic acrylic, silicone, and PMMA IOLs without optic edge classification*. * Each node corresponds to an implant and nodes are connected by an edge if those implants have been compared in RCT. The numbers along the edges, and edge thickness, represent the number of RCTs informing that contrast. RCTs with more than two arms can contribute to multiple contrasts.

**Fig 4:**
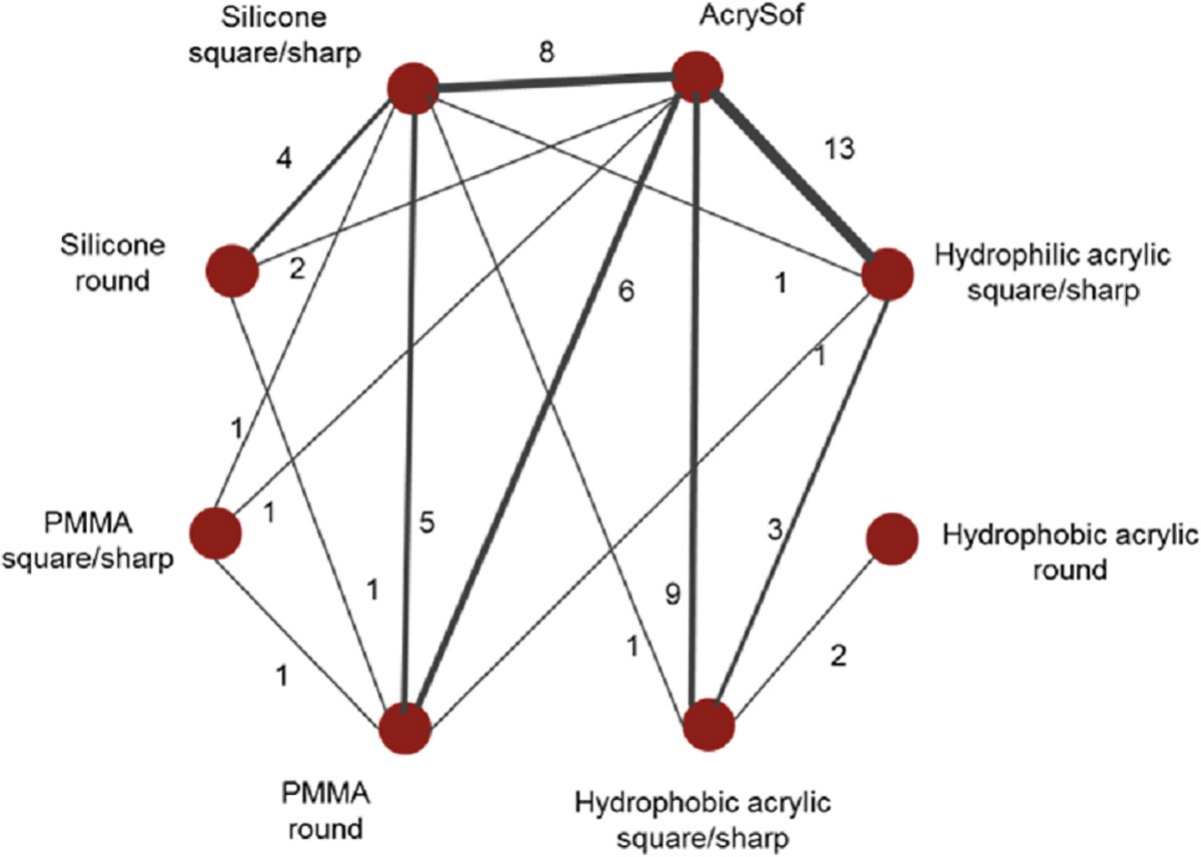
Evidence network for sensitivity analysis: AcrySof vs. other hydrophobic acrylic, hydrophilic acrylic, silicone, and PMMA IOLs with edge classification into round or sharp edged*. * Each node corresponds to an implant and nodes are connected by an edge if those implants have been compared in RCT. The numbers along the edges, and edge thickness, represent the number of RCTs informing that contrast. RCTs with more than two arms can contribute to multiple contrasts.

The evidence networks for the base-case analysis comparing AcrySof material with the other optic materials and for its sensitivity analysis with edge comparison are presented in Figs 3 and 4.

#### Base-case analysis: AcrySof vs other hydrophobic acrylic, hydrophilic acrylic, silicone, and PMMA IOLs without optic edge classification

Data from 37 RCTs were included in the NMA for this contrast. The number of eyes per each IOL group is presented in Table 2. DIC and residual deviance strongly favored the model using only the latest time point from each study and assuming constant HR over time. The residual deviance of this model was 83.4 (95% CrI: 61.1, 110), which is close to the number of data points (n = 80), indicating a good fit to the data. The residual deviance of the inconsistency model was marginally lower at 80.3 (95% CrI: 57.8, 106), but considerable overlap in 95% CrI indicates weak or no evidence of inconsistency. Further model assessment results are provided in the supplement.

**Table 2:** Base-case analysis: Number of eyes in each IOL group.

Treatment IOL	Number of eyes
AcrySof IOL	1947
Hydrophilic acrylic IOL	692
Hydrophobic acrylic IOL	602
PMMA IOL	734
Silicone IOL	755

**Fig 5:**
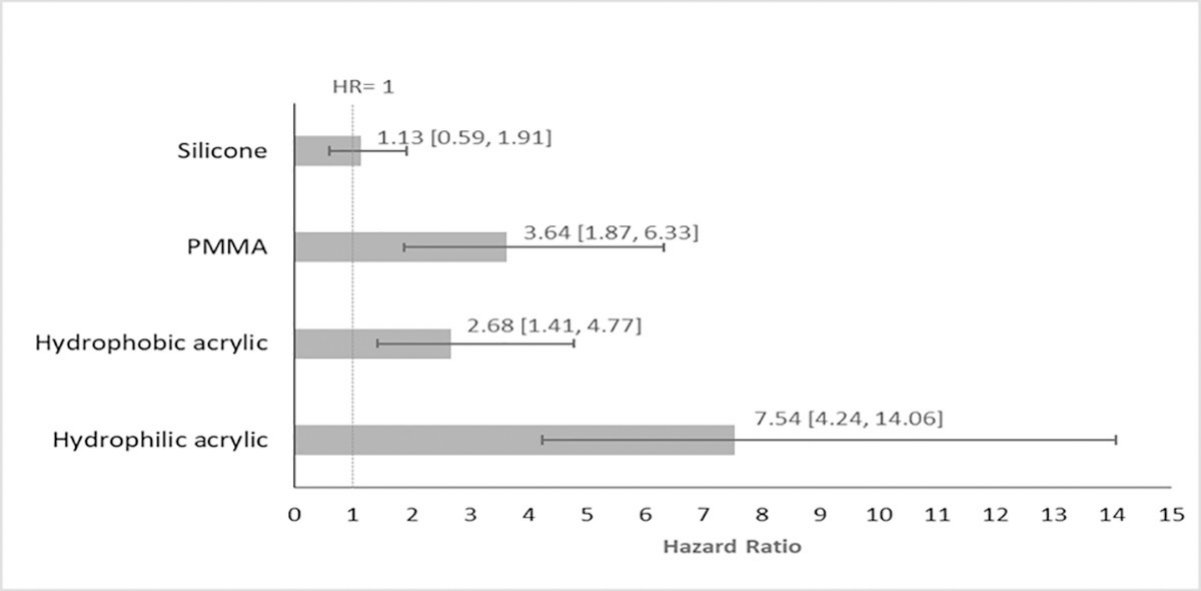
Base-case analysis: Hazard ratios and 95% CrI for Nd:YAG comparing IOL materials vs. AcrySof IOL without optic edge classification*. * Bars represent the hazard ratio relative to AcrySof IOL. Solid lines represent 95% credible intervals.

The mean HR (95% CrI) for Nd:YAG capsulotomy was 1.13 (95% CrI: 0.59, 1.91; p <0.1) with silicone IOLs and 3.64 (95% CrI: 1.87, 6.33; p < 0.001) with PMMA IOLs, compared to AcrySof IOLs. Comparing AcrySof to other acrylic IOLs the mean HR (95% CrI) was 2.68 (95% CrI: 1.41, 4.77; p < 0.01) for other hydrophobic acrylic IOLs and 7.54 (95% CrI: 4.24,14.06; p < 0.001) for hydrophilic acrylic IOLs. AcrySof performed better than all the other IOL materials, (Fig 5).

#### Sensitivity analysis: Sharp edge vs. round edge

Data from 47 RCTs were included in the statistical analysis for sensitivity analysis. The number of eyes per each IOL group is presented in Table 3. As in the base-case, DIC and deviance strongly favored a model using only the latest time point from each study and assuming a constant HR over time. The residual deviance of the selected model using only the latest time point from each study and assuming constant HR over time. The residual deviance of this model was 97.4 (95% CrI: 72.9, 125), which is close to the number of data points (n = 95), indicating a good fit to the data. As for base-case, the residual deviance of the inconsistency model was marginally lower at 94.6 (95% CrI: 70.1, 122), but considerable overlap in CrI again indicates weak or no evidence of inconsistency. Further model assessment results are provided in the supplement.

**Table 3:** Sensitivity analysis: number of eyes in each IOL group.

Treatment IOL	Number of eyes
AcrySof sharp edge IOL	2048
Hydrophilic acrylic sharp edge IOL	692
Hydrophobic acrylic sharp edge IOL	791
PMMA round edge IOL	731
PMMA sharp edge IOL	114
Silicone round edge IOL	420
Silicone sharp edge IOL	743

**Fig 6:**
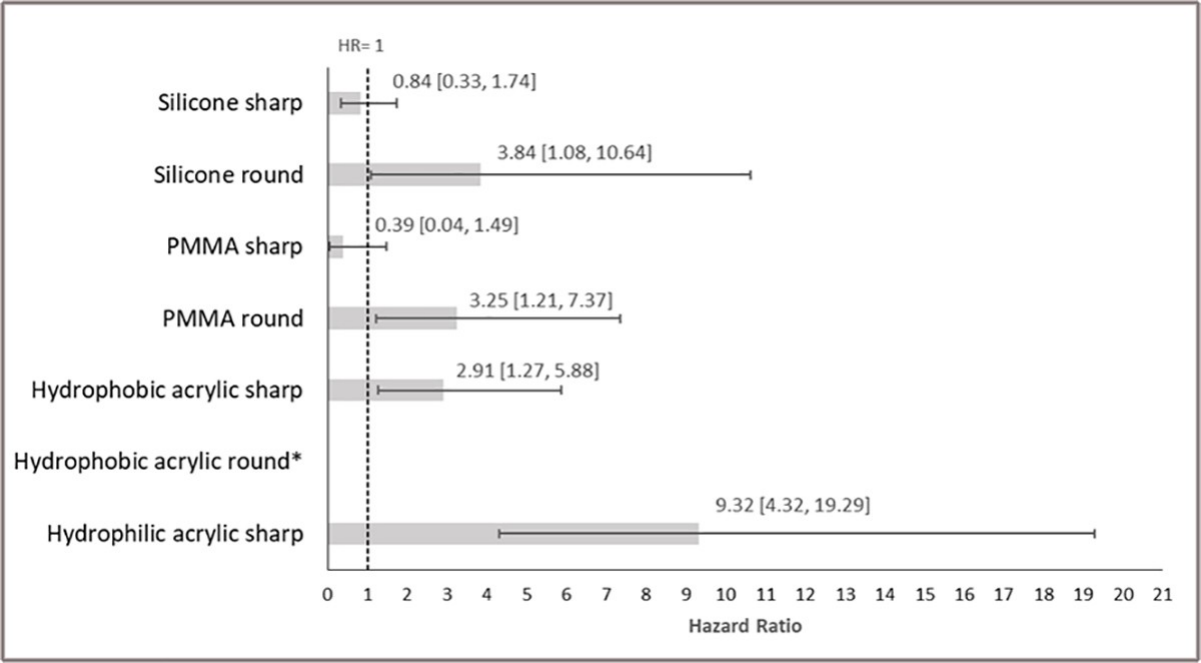
Sensitivity analysis: Hazard ratios and 95% CrI for Nd:YAG comparing IOL materials with sharp/round edge classification vs. AcrySof IOL*. *Bars represent the hazard ratio relative to AcrySof IOL. Solid lines represent 95% credible intervals.

Compared to AcrySof, the mean HR (95% CrI) for Nd:YAG was highest for hydrophilic acrylic sharp-edged IOLs (HR: 9.32; 95% CrI: 4.32, 19.29; p < 0.01), followed by other hydrophobic acrylic sharp-edged IOLs (HR: 2.91; 95% CrI: 1.27, 5.88; p < 0.01), silicone sharp-edged (HR: 0.838; 95% CrI: 0.328, 1.74; p = 0.69), and PMMA sharp-edged (HR: 0.39; 95% CrI: 0.042, 1.49; p = 0.93).Acrysof IOLs had a lower risk of Nd:YAG capsulotomy compared to all round edge IOLs available for analysis: PMMA (HR: 3.25; 95% CrI: 1.21, 7.37; p < 0.01) and silicone (HR: 3.84; 95% CrI: 1.08, 10.64; p < 0.01) (Fig 6).

## Discussion

This NMA evaluated the risk of Nd:YAG capsulotomy associated with different IOL materials and edge designs. Integrating data from the evidence pool of a SLR on the effect of various IOL materials and design on PCO and the incidence of Nd:YAG capsulotomy was essential to understand the comparative effectiveness of treatments and relative ranking of different IOL materials [39]. Such an analysis can provide a simultaneous inference regarding all IOL materials and designs, facilitating the choice of treatment for the treating surgeon [40]. The results of the NMA showed that the risk of Nd:YAG capsulotomy was lower with AcrySof IOL compared to all other IOLs, in the base-case analysis, irrespective of edge properties. The NMA findings strongly favored AcrySof IOLs in comparison to other hydrophobic acrylic IOLs and hydrophilic acrylic IOLs.

In studies by Buehl et al. [41] and Haripriya et al [42], it was observed that the round optic edge of IOL significantly increased the severity of PCO compared to a sharp edge; consequently, the incidence of Nd:YAG was also significantly higher in these IOLs [42]. The sensitivity analysis conducted in the current NMA also highlights the importance of a sharp-edged IOL design in preventing visually significant PCO, requiring Nd:YAG laser capsulotomy.

With respect to edge classification, AcrySof IOL, with sharp optic edge, performed better than all round-edged IOLs. Our findings are similar to the previously conducted meta-analysis of RCTs evaluating the effect of AcrySof IOLs versus PMMA and silicone IOLs on PCO and Nd:YAG capsulotomy rates by Li et al [7] which also reported that AcrySof IOLs had lower Nd:YAG capsulotomy rates than round-edged silicone and PMMA lenses. The effectiveness of hydrophobic acrylic and hydrophilic acrylic IOLs was compared in two recently conducted meta-analyses of RCTs, whereby the reported Nd:YAG capsulotomy rates were lower for the hydrophobic acrylic group [8, 13]. Furthermore, in a head-to-head meta-analysis, AcrySof IOLs were associated with a significantly lower risk of Nd:YAG capsulotomy compared to other acrylic IOLs [43]. Unlike an NMA, these meta-analyses included only direct evidence for their comparison. Further to evidence from RCTs, similar results from observational research have been reported. In Ursell et al (2018), the authors assess AcrySof versus groups of other hydrophobic and hydrophilic IOLs, concluding that the risk of undergoing an Nd:YAG capsulotomy is significantly lower when AcrySof IOLs are used [43].

It has been commonly reported that hydrophilic acrylic IOLs are associated with a higher incidence of Nd:YAG capsulotomy compared to hydrophobic acrylic IOLs, attributed to different material properties and relatively duller edges of hydrophilic IOLs [24, 25, 44]. Although all hydrophilic IOLs included in the present analysis are categorized as having a sharp edge optic design, these lenses are machined in a dehydrated state and then hydrated. It has been posited that as a result of the swelling of the IOLs during hydration, the optic edges could become less sharp, potentially removing the barrier effect [45]. Further to this, hydrophobic acrylic IOLs can adhere to the collagen membrane of LECs, leading to tight apposition of IOLs in posterior capsular bag, and advanced adhesiveness through fibronectin [46, 47]. This may result in less space between the IOL and the posterior capsule where the LECs could migrate [8]. By contrast, hydrophilic acrylic materials have been shown to promote LEC adhesion, migration, and proliferation and consequently, PCO development [48].

In the present analysis, AcrySof IOLs, which are made from a hydrophobic acrylic material with a sharp edge optic design, are found to be associated with a lower risk of Nd:YAG capsulotomy and by proxy a lower rate of visually significant PCO–compared to a group of other hydrophobic acrylic IOLs, also with a sharp edge design. It has been suggested that these differences could be attributed to the sharper optic edge profile of AcrySof IOLs compared to other hydrophobic IOLs [49]. However, in Leydolt et al. 2013, the AcrySof SN60WF IOL displayed lower PCO and Nd:YAG capsulotomy rates compared to the Hoya iMics1 NY-60 IOL, despite the Hoya lens having a sharper optic edge than AcrySof SN60WF [50]. This led the authors to conclude that it is not only a sharp optic edge but also the characteristics of the individual lens material that play an important role in the prevention of PCO. Further to this, it has been found that the acrylate material used in AcrySof IOLs shows superior fibronectin binding compared to other hydrophobic materials, this may also offer a rationale for the lower PCO and subsequent Nd:YAG rates associated with AcrySof IOLs [51].

It has been previously noted that sharp-edged PMMA and silicone IOLs exhibit good PCO preventing properties [5]. This is consistent with the results of the present study–whereby, sharp-edge PMMA and silicone IOLs perform comparably to AcrySof in terms of reducing the incidence of Nd:YAG capsulotomy. However, it is the case that these IOL materials are infrequently used in current clinical practice [5]. The implantation of PMMA IOLs require a larger wound size which can be a concern with respect to post-operative astigmatism, pupil ovalization and endothelial cell loss [5, 52]. Additionally, it has been reported that plate haptic silicone IOLs can dislocate posteriorly following Nd:YAG capsulotomy due to spontaneous capsular contraction [53].

Acrylic IOLs have become the standard of care for patients undergoing cataract surgery, among which hydrophobic acrylic are predominant in use in the western world [6]. As per the European Registry of Quality Outcomes for Cataract and Refractive Surgery (EURQUO) guidelines, hydrophobic acrylic IOLs accounted for 80.8% of implants used in cataract surgery, while hydrophilic acrylic and silicone IOLs accounted for 14% and 3.5%, respectively [6]. However this study provides evidence that within the dominating group of hydrophobic acrylic IOLs significant differences exist as it is evident by the superior results of AcrySof versus the group of non-Acrysof hydrophobic acrylic IOLs evaluated.

As previously discussed, cataract surgery is one of the most commonly reported surgical procedures world-wide. Indeed in the US, cataract surgery with IOL implantation was the most commonly performed surgical procedure and the single largest expenditure for any Part B procedure in the Medicare program. In 2010 (latest year available), payment for cataract was $2 billion, which was 1.8% of total allowed charges. Additionally, and for the same year–the costs associated with the Nd:YAG capsulotomy procedure amounted to $187.5 million [17]. Considering the magnitude of the reported costs, reducing the incidence of Nd:YAG capsulotomy to correct PCO via the implantation of the most appropriate choice of IOL, is likely to translate to significant savings for national healthcare systems–while at the same time reducing the clinical risks associated with the procedure.

The current SLR has strengths and limitations. The SLR includes moderate quality RCTs published in a period of more than 20 years (1996 –August 2017). Despite restricting to only English language studies, 67 publications from 59 RCTs were identified and formed an evidence base suitable for an NMA. However, RCTs with high or unclear risk of selection bias were also included at the time of quality assessment in the interest of constructing a comprehensive evidence network. While information about randomization was not explicitly mentioned in these trials, it may be safe to assume that at least a simple randomization method was adopted. It is the case that blinding is more difficult to perform in medical device trials compared to pharmacological trials [54]. While blinding of surgeons many not always be possible, blinding of outcome assessors can be carefully planned [54]. In the SLR, it was observed that severity of PCO is a more frequently reported outcome than the incidence rate of PCO itself. Severity of PCO is assessed and reported as mean severity scores, using various objective and subjective scales. Scales such as AQUA or Automated Quantification of After-Cataract (scale of 0–10), and EPCO or Evaluation of Posterior Capsule Opacification (scale of 0–4) are objective systems used to analyze the digitally acquired retroillumination photographs of the posterior capsule [4]. Another system known as the POCO-MAN calculates the percentage of capsule with opacification placing a software grid over the digital image. Additionally, there are several scales subjectively grading PCO on a scale of 1–10 using slit-lamp images [4, 55]. Each system has its own benefits and limitations. In previous meta-analyses by Li et al. [7, 8], an attempt was made to account for different scales across the evaluation systems using standardized mean differences. However, the synthesis of such a heterogeneous variable using NMA methodology was beyond the scope of the present study.

Our network meta-analysis also has several strengths and limitations. The model selected used only the latest time point from each study and made the potentially unrealistic assumption of constant HR (relative Nd:YAG rates) over time, but we explored a range of alternative modelling approaches. These included models that utilized multiple reported follow-up times per study and models that made piecewise constant assumptions about the relative Nd:YAG rates [31]. However, these alternative models had inferior model assessment statistics compared with our selected model and all fit poorly to the data. Inconsistency was also a potential problem in our selected model for both evidence networks. Although the test revealed weak or no evidence of inconsistency, these tests are inherently underpowered and inconsistency may be an issue, as in all NMAs [56, 57].

Further to this, this research focusses solely on published evidence from RCTs. Observational data would likely also be valuable to inform this research question–further exploration of same may be warranted in future research.

## Conclusions

Our study reaffirms that lens material and a sharp optic edge design play an important role in reducing Nd:YAG capsulotomy rates. The NMA presents strong evidence to suggest that the risk of Nd:YAG capsulotomy is lower in eyes implanted with AcrySof IOLs compared to non-AcrySof hydrophobic or hydrophilic acrylic IOLs. The appropriate choice of IOL implanted during cataract surgery is an important consideration to reduce the risk of Nd:YAG laser capsulotomy in the post-surgery period and may translate into significant savings for national healthcare systems while at the same time reducing the clinical risks associated with the procedure.

## Supporting information

S1 File Additional details on network meta-analysis. (DOCX)

S2 File Prisma checklist. (DOCX)

S3 File Additional details on Publication bias assessment. (ZIP)

S4 File Minimal data set. (XLSX)
