# Supplementary material for: Effect of AcrySof versus other intraocular lens properties on the risk of Nd:YAG capsulotomy after cataract surgery: A systematic literature review and network meta-analysis
Source: PLoS One. 2019 Aug 19;14(8):e0220498. doi: 10.1371/journal.pone.0220498 (PMC6699683; doi:10.1371/journal.pone.0220498)
Supplement: S1 File — (DOCX) [file pone.0220498.s001.docx]

**S1 Supporting information**

**Additional details on network meta-analysis**

The notation for our models is as follows. We have $N_{s}$ studies with ${NA}_{j}$ arms for study $j$ and, possibly, multiple observations (follow-ups $F$) for each of these arms. We label these observations $i\in1,\ldots,N_{obs}$ where observation $i$ is from study $s_{i}$. For arm $k$ of observation $i$ we have the number of Nd:YAG events $r_{ik}$ out of $n_{ik}$ patients. Each of these observations has a time at risk $F_{ik}$, which is the time in months from the previous to the current observation. For example, a two-arm trial with only one observation at 12 months follow-up will have $F_{i1}=F_{i2}=12$. We considered six models for this data but, following model comparison, selected model M3 as our base case.

**M1. Multiple observations per study, cloglog link, shared baseline within studies**

As the Nd:YAG event cannot repeat (patients leave the trial on experiencing the event) but the rate increases for later follow-up, we follow a binomial likelihood with complementary log-log link (28). We assume a binomial likelihood

$$r_{ik}\sim binomial(p_{ik},n_{ik})$$

With a complementary log-log link connecting the probability $p_{ik}$ to the log hazard scale.

$$\mathrm{cloglog}\left( p_{ik} \right)=log \left( -\log\left( p_{ik} \right) \right)=\log\left( F_{ik} \right)+\mu_{s_{i}}+\delta_{s_{i}k}I_{k\neq b}$$

This implies an exponential failure model $p_{ik}=1-\exp\left( -\lambda_{ik}F_{ik} \right)$ with $\log\left( \lambda_{ik} \right)=\mu_{s_{i}}+\delta_{s_{i}k}I_{k\neq b}$. The baseline effect $\mu_{s_{i}}$ is the log hazard of Nd:YAG for study $s_{i}$, assumed constant across reported follow-ups of the trial. This would imply the same $\mu_{s_{i}}$ at 6 and 200 months, which may not be realistic. The $\log\left( F_{ik} \right)$ is the log of follow-up time, which acts as an offset. It is time at which patients in arm $k$ of study $s_{i}$ are exposed to total log hazard $\mu_{s_{i}}+\delta_{s_{i}k}$

The $\delta_{s_{i}k}$ is the log hazard ratio of Nd:YAG for patients on the implant in arm $k$ relative to the implant in control arm $b$. We attempted two modelling assumptions

- Random effects model: $\delta_{s_{i}k}\sim Normal\left( d_{t_{k}}-d_{t_{b}},\sigma^{2} \right)$
- Fixed effects model: $\delta_{s_{i}k}=$ $d_{t_{s_{i}k}}-d_{t_{s_{i}b}}$

Where $d_{a}$ is the log hazard ratio of Nd:YAG for treatment $t=a$ relative to the reference treatment of the NMA (labelled $t=1)$. We chose random effects and do not report the fixed effects results as convergence was very poor, likely due to the unrealistic assumption of common log hazard ratios at different follow-up times. Note that the (possibly unrealistic) assumption of constant hazard ratios is made as the same $\delta_{s_{i}k}$ applies at each follow-up $F_{\cdot\cdot}$ reported by the study. It further makes the assumption that the log hazard ratios relative to the reference $d_{a}$ are constant over time.

**M2. Multiple observations per study, cloglog link, independent baselines within studies**

Our second model for these observations also assumes a binomial likelihood

$$r_{ik}\sim binomial(p_{ik},n_{ik})$$

And uses a complementary log-log link to the log hazard scale.

$$\mathrm{cloglog}\left( p_{ik} \right)=log \left( -\log\left( 1-p_{ik} \right) \right)=\log\left( F_{ik} \right)+\mu_{i}+\delta_{s_{i}k}I_{k\neq b}$$

The difference is that now the baseline effect $\mu_{i}$ is the log hazard of Nd:YAG for each observation $i$, which is different for each reported follow-up of study $s_{i}$. This allows different $\mu_{i}$ at 6 and 200 months, which may be more realistic. Although this model may be more flexible than the shared nuisance parameter, it requires more data to fit.

The $\delta_{s_{i}k}$ is again the log hazard ratio of Nd:YAG for patients on the implant in arm $k$ relative to the implant in control arm $b$. We again assume a random effects model ($\delta_{s_{i}k}\sim Normal\left( d_{t_{k}}-d_{t_{b}},\sigma^{2} \right)$) due to poor convergence of the fixed effects model. These log hazard ratios $d_{a}$ relative to the reference implant are again assumed constant over time. The $\log\left( F_{ik} \right)$ is the log of follow-up time, which acts as an offset; it is time at which patients in arm $k$ of observation $i$ are exposed to total log hazard $\mu_{i}+\delta_{s_{i}k}$.

**M3 (base-case analysis). Latest timepoint only, cloglog link.**

This model differs from M1 and M2 in that only one observation is used per study so $s_{i}=i$. The model is again binomial likelihood

$$r_{ik}\sim binomial(p_{ik},n_{ik})$$

with a complementary log-log link

$$\mathrm{cloglog}\left( p_{ik} \right)=log \left( -\log\left( p_{ik} \right) \right)=\log\left( F_{ik} \right)+\mu_{i}+\delta_{s_{i}k}I_{k\neq b}$$

Note that this is identical to model M2 and, in fact, the code for either M1 or M2 applied to this data will give the same result as $\mu_{s_{i}}=\mu_{i}$. The $\delta_{s_{i}k}$ is again the log hazard ratio of Nd:YAG for patients on the implant in arm $k$ relative to the implant in control arm $b$. The log hazard ratios $d_{a}$ relative to the reference implant are again assumed constant over time. The same log hazard applies no matter the $F_{ik}$ of the observation.

**Piecewise constant models (M1pc, M2pc, M3pc)**

Instead of assuming a constant log hazard ratio $d_{a}$ (relative to reference implant) over time, we now assume piecewise constant log hazard ratios $d_{1a}$, $d_{2a}$, and $d_{3a}$. This model can be fit to data with single or multiple time points reported per study. Lu and Ades have explored such models in detail (29) and they have been used in literature (63).

The model can be fit with either shared baselines $\mu_{s_{i}}=\mu_{i}$ (M1pc), independent baselines (M2pc), or with only the latest timepoint from each study (M3pc). We describe the shared baseline M1pc but independent M2pc is similar while M3pc uses the same model but includes only the latest timepoint from each study. We assume the follow-up time across all studies is split into three categories: 0-1 year, 1-2 years, 2-10 years, as have been used in previous meta-analyses (10). The likelihood is binomial (single Nd:YAG event per patient)

$$r_{ik}\sim binomial(p_{ik},n_{ik})$$

With a complementary log-log link

$$\mathrm{cloglog}\left( p_{ik} \right)=log \left( -\log\left( p_{ik} \right) \right)=\log\left( f_{1ik}\lambda_{1ik}+f_{2ik}\lambda_{2ik}+f_{3ik}\lambda_{3ik} \right)$$

The $f_{1ik}$ is the amount of follow-up $F_{ik}$ that occurs in the first period, $f_{2ik}$ is amount in second period, and $f_{3ik}$ in third. For example, a follow-up from 6 months to 24 months gives $F_{ik}=18$ but $f_{1ik}=6$, $f_{2ik}=12$, and $f_{3ik}=0$. In this model it is important *when* the follow-up takes place, not only its duration.

The hazard $\lambda_{lik}$ in period $l$for arm $k$ of observation $i$ (of study $s_{i}$) is then

$$\lambda_{lik}=\mu_{s_{i}}+\delta_{ls_{i}k}I_{k\neq b}$$

With a random effects model for each of the three periods (note the shared heterogeneity variance $\sigma^{2}$ across time periods):

$$\delta_{{ls}_{i}k}\sim Normal\left( d_{{lt}_{k}}-d_{lt_{b}},\sigma^{2} \right)$$

Or a fixed effects model (which may fit better now that follow-up times are being categorised):

$$\delta_{{ls}_{i}k}=d_{{lt}_{k}}-d_{lt_{b}}$$

The log hazard ratios $d_{la}$ for treatment $t=a$ are now specific to each of the three time periods $l=1,2,3$. We assume$d_{la}\sim Normal(d_{a},\omega^{2})$, giving a single treatment effect $d_{a}$ that is a shared mean across time periods.

**Model comparison results.**

The six models, all assuming random treatment effects, were run using OpenBUGS version 3.2.3. Model comparison statistics for contrasts 1 and 2 are presented below. The Deviance Information Criterion (DIC) is a model assessment statistic that contrasts the fit and complexity of models while total residual deviance is a assessment only of model fit (30). Lower values are preferred for both DIC and deviance but they can only be compared across models that share the same data (number of data points below). The total residual deviance can be compared with the number of data points to provide an overall assessment of model fit.

Table S1 presents results of this model comparison. The residual deviance strongly favors model M3, which uses only the latest time point from each study. Although piecewise constant models improve the DIC and deviance of models using multiple time points per study, their deviance still suggests poor fit. Only results from model M3 are presented in the main text as deviance of the alternative models was judged too high for them to give reliable results.

**Table S1. Comparison of models fit to the Nd:YAG base-case and sensitivity analyses evidence networks**

|  | **Base-case analysis** | | | **Sensitivity analysis** | | |
| --- | --- | --- | --- | --- | --- | --- |
|  | **DIC*** | **Total residual deviance** | **Number of data points** | **DIC*** | **Total residual deviance** | **Number of data points** |
| **M1** | 792.1 | 263 (241, 290) | 102 | 873.7 | 293 (269, 321) | 125 |
| **M2** | 681.9 | 167 (143, 195) | 102 | 772.9 | 186 (160, 218) | 125 |
| **M3** | 499.2 | 83.4 (61.1, 110) | 80 | 572.3 | 97.4 (72.9, 125) | 95 |
| **M1pc** | 731.7 | 145 (117, 178) | 102 | 731.7 | 178 (148, 212) | 125 |
| **M2pc** | 615.9 | 144 (117, 174) | 102 | 737.5 | 179 (150, 213) | 125 |
| **M3pc** | 503.7 | 115 (89.2, 144) | 80 | 503.7 | 132 (104, 164) | 95 |

* DIC can be compared only across models sharing datasets; namely M1, M2, M1pc, M2pc on base-case and the same models on sensitivity analysis

**Inconsistency assessment**

Inconsistency is disagreement between direct and indirect evidence on a treatment comparison. It can be assessed whenever there are closed loops in the evidence networks; as there are for both base-case analysis and sensitivity analysis. We explored inconsistency in model M3 by fitting an inconsistency model (32) which assumes changes the underlying random treatment effect model to

$$\delta_{s_{i}k}\sim Normal\left( d_{t_{b}t_{k}},\sigma^{2} \right)$$

With the $d_{uv}$ log hazard ratios all assumed independent.

The results of the inconsistency assessment are presented in Table X2 below. Both the DIC and deviance are marginally lower in the inconsistency model but the 95% credible intervals are entirely overlapping. This suggests limited or no evidence of inconsistency in the network. However, it should be noted that inconsistency assessments have low power and a failure to detect inconsistency does not assure that there is no inconsistency in the network.

**Table S2. Inconsistency assessment results for model with latest time point only (M3) on base-case and sensitivity analysis**

| **Contrast** | **Model** | **DIC** | **Residual Deviance** | **Number of data points** |
| --- | --- | --- | --- | --- |
| Base-case analysis | M3 consistency | 572.3 | 83.4 (61.1, 110) | 80 |
|  | M3 inconsistency | 570.5 | 80.3 (57.8, 106) | 80 |
| Sensitivity analysis | M3 consistency | 499.2 | 97.4 (72.9, 125) | 95 |
|  | M3 inconsistency | 497.5 | 94.6 (70.1, 122) | 95 |
