# Supplementary material for: Effect of AcrySof versus other intraocular lens properties on the risk of Nd:YAG capsulotomy after cataract surgery: A systematic literature review and network meta-analysis
Source: PLoS One. 2019 Aug 19;14(8):e0220498. doi: 10.1371/journal.pone.0220498 (PMC6699683; doi:10.1371/journal.pone.0220498)
Supplement: S2 File — (DOCX) [file pone.0220498.s002.docx]

**S2 Supporting information**

**Table 1 S2: PRISMA Checklist**

| **Section/topic** | **#** | **Checklist item** | **Reported on page #** |
| --- | --- | --- | --- |
| **TITLE** | | |  |
| Title | 1 | Identify the report as a systematic review, meta-analysis, or both. | 1 |
| **ABSTRACT** | | |  |
| Structured summary | 2 | Provide a structured summary including, as applicable: background; objectives; data sources; study eligibility criteria, participants, and interventions; study appraisal and synthesis methods; results; limitations; conclusions and implications of key findings; systematic review registration number. | 2 |
| **INTRODUCTION** | | |  |
| Rationale | 3 | Describe the rationale for the review in the context of what is already known. | 5/6 |
| Objectives | 4 | Provide an explicit statement of questions being addressed with reference to participants, interventions, comparisons, outcomes, and study design (PICOS). | 7 |
| **METHODS** | | |  |
| Protocol and registration | 5 | Indicate if a review protocol exists, if and where it can be accessed (e.g., Web address), and, if available, provide registration information including registration number. | n/a |
| Eligibility criteria | 6 | Specify study characteristics (e.g., PICOS, length of follow-up) and report characteristics (e.g., years considered, language, publication status) used as criteria for eligibility, giving rationale. | 7 |
| Information sources | 7 | Describe all information sources (e.g., databases with dates of coverage, contact with study authors to identify additional studies) in the search and date last searched. | 10 |
| Search | 8 | Present full electronic search strategy for at least one database, including any limits used, such that it could be repeated. | 4 (S2) |
| Study selection | 9 | State the process for selecting studies (i.e., screening, eligibility, included in systematic review, and, if applicable, included in the meta-analysis). | 10 |
| Data collection process | 10 | Describe method of data extraction from reports (e.g., piloted forms, independently, in duplicate) and any processes for obtaining and confirming data from investigators. | 7/8 |
| Data items | 11 | List and define all variables for which data were sought (e.g., PICOS, funding sources) and any assumptions and simplifications made. | 8/9 |
| Risk of bias in individual studies | 12 | Describe methods used for assessing risk of bias of individual studies (including specification of whether this was done at the study or outcome level), and how this information is to be used in any data synthesis. | 10 |
| Summary measures | 13 | State the principal summary measures (e.g., risk ratio, difference in means). | 2 |
| Synthesis of results | 14 | Describe the methods of handling data and combining results of studies, if done, including measures of consistency (e.g., I^2^) for each meta-analysis. | 9 |

Page 1 of 2

| **Section/topic** | **#** | **Checklist item** | **Reported on page #** |
| --- | --- | --- | --- |
| Risk of bias across studies | 15 | Specify any assessment of risk of bias that may affect the cumulative evidence (e.g., publication bias, selective reporting within studies). | 10 |
| Additional analyses | 16 | Describe methods of additional analyses (e.g., sensitivity or subgroup analyses, meta-regression), if done, indicating which were pre-specified. | 8 |
| **RESULTS** | | |  |
| Study selection | 17 | Give numbers of studies screened, assessed for eligibility, and included in the review, with reasons for exclusions at each stage, ideally with a flow diagram. | 10 |
| Study characteristics | 18 | For each study, present characteristics for which data were extracted (e.g., study size, PICOS, follow-up period) and provide the citations. | 11 (S2) |
| Risk of bias within studies | 19 | Present data on risk of bias of each study and, if available, any outcome level assessment (see item 12). | 5 (S2) |
| Results of individual studies | 20 | For all outcomes considered (benefits or harms), present, for each study: (a) simple summary data for each intervention group (b) effect estimates and confidence intervals, ideally with a forest plot. | 11-15 |
| Synthesis of results | 21 | Present results of each meta-analysis done, including confidence intervals and measures of consistency. | 11-15 (8 SI) |
| Risk of bias across studies | 22 | Present results of any assessment of risk of bias across studies (see Item 15). | 10 |
| Additional analysis | 23 | Give results of additional analyses, if done (e.g., sensitivity or subgroup analyses, meta-regression [see Item 16]). | 12-15 |
| **DISCUSSION** | | |  |
| Summary of evidence | 24 | Summarize the main findings including the strength of evidence for each main outcome; consider their relevance to key groups (e.g., healthcare providers, users, and policy makers). | 15-20 |
| Limitations | 25 | Discuss limitations at study and outcome level (e.g., risk of bias), and at review-level (e.g., incomplete retrieval of identified research, reporting bias). | 19/20 |
| Conclusions | 26 | Provide a general interpretation of the results in the context of other evidence, and implications for future research. | 21 |
| **FUNDING** | | |  |
| Funding | 27 | Describe sources of funding for the systematic review and other support (e.g., supply of data); role of funders for the systematic review. | Entered into online system |

*From:*  Moher D, Liberati A, Tetzlaff J, Altman DG, The PRISMA Group (2009). Preferred Reporting Items for Systematic Reviews and Meta-Analyses: The PRISMA Statement. PLoS Med 6(7): e1000097. doi:10.1371/journal.pmed1000097

**Table 2 S2: Search strategy**

**Medline and Embase through Embase.com**

| No. | Search string | # of hits |
| --- | --- | --- |
| #1 | **'cataract'**/exp | 50656 |
| #2 | **'cataract extraction'**/exp | 40114 |
| #3 | **'phacoemulsification'**/exp | 11104 |
| #4 | ((**extract*** OR **aspirat*** OR **operat*** OR **remov*** OR **surg*** OR **excis*** OR **implant***) NEAR/3 (**cataract*** OR **lens***)):ab,ti | 34371 |
| #5 | **'lens implantation'**/de | 6589 |
| #6 | **'lens implant'**/de | 18784 |
| #7 | **'pseudophakia'**/de | 3941 |
| #8 | ((**intraocular** OR **'intra ocular'** OR **'intra-ocular'**) NEXT/1 **lens***):ab,ti OR **iol***:ab,ti | 19053 |
| #9 | **#1** OR **#2** OR **#3** OR **#4** OR **#5** OR **#6** OR **#7** OR **#8** | 90468 |
| #10 | **'clinical trial'**/exp OR **'randomized controlled trial'**/de OR **'randomization'**/de OR **'single blind procedure'**/de OR **'double blind procedure'**/de OR **'crossover procedure'**/de OR **'placebo'**/de | 1307578 |
| #11 | **'randomized controlled trial'**:ab,ti OR **rct**:ab,ti OR **'randomised controlled trial'**:ab,ti | 91207 |
| #12 | (**random*** NEAR/3 **allocat***):ab,ti OR **random*** | 1258016 |
| #13 | ((**single** OR **double** OR **treble** OR **triple**) NEAR/3 **blind**):ab,ti | 170569 |
| #14 | **placebo***:ab,ti | 238533 |
| #15 | **'prospective study'**/de | 330399 |
| #16 | **#10** OR **#11** OR **#12** OR **#13** OR **#14** OR **#15** | 2231108 |
| #17 | **'case study'**/de OR **'case report'**:ab,ti OR **'letter'**/de OR **'review'**/de OR **commentary** OR **'editorial'**/de | 3926777 |
| #18 | **#16** NOT **#17** | 1898044 |
| #19 | **'clinical study'**/de OR **'case control study'** OR **'longitudinal study'**/de OR **'open study'**/de OR **'major clinical study'**/de OR **'retrospective study'**/de OR **'observational study'**/de | 3083604 |
| #20 | **'prospective study'**/de | 330399 |
| #21 | **'randomized controlled trials'**/de | 99662 |
| #22 | **#20** NOT **#21** | 327566 |
| #23 | **cohort** NEXT/1 (**study** OR **studies**) | 168051 |
| #24 | ((**'case control'** OR **'follow up'** OR **observational** OR **epidemiologic*** OR **'cross sectional'**) NEXT/1 (**study** OR **studies**)):ab,ti | 433919 |
| #25 | **#19** OR **#22** OR **#23** OR **#24** | 3492138 |
| #26 | **#18** OR **#25** | 4658451 |
| #27 | **'neodymium yag laser'**/exp OR **'neodymium yag laser'**/syn OR **'nd-yag'**:ab,ti OR **'nd yag'**:ab,ti OR **'nd:yag'**:ab,ti OR **'neodymium:yttrium-aluminum-garnet'**:ab,ti OR **'posterior capsular opacification'** OR **capsulotom***:ab,ti OR **pco**:ab,ti OR (**posterior** NEAR/3 **opac***):ab,ti | 27065 |
| #28 | **#9** AND **#26** AND **#27** AND [humans]/lim | 1513 |

**Table 3 S2: Risk of bias assessment**

| **Study name** | **Was randomisation carried out appropriately?** | **Was the concealment of treatment allocation adequate?** | **Were the groups similar at the outset of the study in terms of prognostic factors?** | **Were the care providers, participants and outcome assessors blind to treatment allocation?** | **Were there any unexpected imbalances in dropouts between groups?** | **Is there any evidence to suggest that the authors measured more outcomes than they reported?** | **Did the analysis include an intentionto- treat analysis? If so, was this appropriate and were appropriate methods used to account for missing data?** |
| --- | --- | --- | --- | --- | --- | --- | --- |
| Chang 2017 | Yes | Yes | Yes | Yes | N.A | No | No |
| Haripriya 2017 | Yes | Unclear | N.A | Yes | Yes | No | No |
| Johansson 2017 | Yes | Unclear | N.A | Unclear | N.A | No | No |
| Kahraman 2017 | Yes | Yes | N.A | Yes | N.A | No | No |
| Leydolt 2017 | Yes | Yes | N.A | Yes | N.A | No | No |
| Kalauz 2016 | Unclear | Unclear | Yes | No | Yes | No | No |
| Nagata 2015 | Unclear | Unclear | Unclear | Unclear | Unclear | No | No |
| Findl 2015 | Yes | Yes | N.A | Yes | N.A | N.A | No |
| Kahraman 2015 | Unclear | Yes | N.A | No | N.A | No | No |
| Krall 2015 | Unclear | Unclear | N.A | Yes | No | No | No |
| Schriefl 2015 | Yes | Unclear | Yes | Unclear | Unclear | No | Yes |
| Schriefl 2015 | Yes | Unclear | Yes | Unclear | Unclear | No | Yes |
| Kahraman 2014 | Unclear | Yes | N.A | No | N.A | No | No |
| Mylonas 2014 | Unclear | Unclear | N.A | Yes | Unclear | No | No |
| Ronbeck 2014 (Ronbeck 2009, Wegde 2004, Wegde 2003) | Yes | Unclear | Yes | Unclear | Yes | No | No |
| Chang 2013 | Unclear | Unclear | Yes | No | No | N.A | Unclear |
| Hirnschall 2013 | Yes | Yes | N.A | Yes | Unclear | No | No |
| Leydolt 2013 | Yes | Yes | N.A | Yes | Unclear | No | No |
| Leydolt and Schriefl 2013 (NCT01732484) | Yes | Yes | N.A | Yes | N.A | No | No |
| Mylonas 2013 (NCT 00673803) | Unclear | Unclear | N.A | Yes | N.A | No | No |
| Nanavaty 2013 | Yes | Unclear | N.A | No | N.A | No | No |
| Prinz 2013 | Yes | Yes | Yes | Yes | No | No | No |
| Nanavaty 2012 (NCT00762021) | Yes | Unclear | N.A | No | N.A | No | Yes |
| Gangwani 2011 | Yes | Yes | N.A | Yes | N.A | N.A | No |
| Iwase 2011 | Unclear | Unclear | N.A | Unclear | N.A | No | Yes |
| Vasavada 2011 | Yes | Unclear | Yes | No | N.A | No | No |
| Zemaitiene 2011 | Unclear | Unclear | Yes | No | No | No | No |
| Cleary 2009 | Yes | No | N.A | No | N.A | N.A | No |
| Vock 2009 | Yes | Unclear | N.A | Yes | N.A | No | Yes |
| Hayashi 2008 | Yes | Yes | N.A | Yes | No | N.A | Yes |
| Kang 2008 | Unclear | Unclear | Yes | Unclear | No | No | Yes |
| Kohnen 2008 (Linked: Mester 2004) | Yes | Unclear | Yes | No | N.A | No | No |
| Kugelberg 2008 (Kugelberg 2006) | Yes | Yes | Yes | Yes | Unclear | No | No |
| Barisic 2007 | Unclear | Unclear | Yes | No | No | N.A | Unclear |
| Buehl 2007 (Linked: Buehl 2004) | Unclear | Unclear | N.A | Yes | No | N.A | Yes |
| Hancox 2007 | Unclear | Unclear | N.A | No | Yes | N.A | No |
| Hayashi 2007 | Yes | Yes | N.A | Yes | No | N.A | Yes |
| Kaya 2007 | Yes | Unclear | Yes | Unclear | No | No | Yes |
| Leydolt 2007 | Unclear | Unclear | N.A | Yes | Unclear | No | No |
| Vock 2007 | Unclear | Unclear | N.A | No | N.A | No | No |
| Zemaitiene 2007 | Unclear | Unclear | Yes | No | Yes | No | No |
| Buehl 2002 (Linked: Buehl 2005) | Unclear | Unclear | N.A | Yes | Yes | N.A | No |
| Findl 2005 | Yes | Yes | N.A | Yes | N.A | N.A | No |
| Hayashi 2005 | Yes | Yes | N.A | Yes | No | N.A | Yes |
| Heatly 2005 | Unclear | Unclear | N.A | No | No | N.A | No |
| Menapace 2005 | Unclear | Unclear | Unclear | Yes | Unclear | No | No |
| Sacu 2005 | Unclear | Unclear | Yes | Yes | N.A | No | No |
| Sundelin-2005 (Linked: Sundelin-2006) | Unclear | Unclear | Yes | No | Yes | No | No |
| Hayashi 2004 | Yes | Yes | N.A | Yes | Yes | N.A | Yes |
| Sacu 2004 | Unclear | Unclear | Yes | Yes | N.A | No | No |
| Sacu 2004 | Unclear | Unclear | Yes | Yes | Unclear | No | No |
| Ernest 2003 | Unclear | Unclear | N.A | No | N.A | N.A | Yes |
| Hayashi 2001 | Yes | Yes | Yes | Yes | No | N.A | No |
| Hollick 2000 | Yes | Unclear | Yes | Yes | Unclear | No | No |
| Kobayashi 2000 | Yes | Unclear | Yes | Unclear | Unclear | No | No |
| Kucuksumer 2000 | Yes | Unclear | N.A | Yes | N.A | No | No |
| Wang 2000 | Unclear | Unclear | Yes | No | No | No | No |
| Hollick 1999 | Yes | Unclear | Yes | Unclear | Unclear | No | No |
| Hayashi 1998 | Unclear | Yes | Yes | No | Yes | N.A | No |
| Ursell 1998 | Yes | Unclear | Unclear | Unclear | Unclear | No | No |

**Table 3 S2: Overview of studies included for systematic review**

| Study name (Author, year) or Trial acronym or NCT | Location (Country) | Study period (month, year) | Study sample (patients, no. of eyes) | Follow-up (as reported) | IOL comparison | Outcomes assessed |
| --- | --- | --- | --- | --- | --- | --- |
| Chang 2017 | Sweden | May 2002 to March 2004 | 120 patients | 9 years | AcrySof SA60AT vs. BL27 | Incidence of Nd:YAG capsulotomy |
| Haripriya 2017 (NCT00312299) | India | 2006 to 2016 | 100 patients (200 eyes) | 9 years | S3602 SQ (square edge) vs. S3602 (round edge) vs. AcrySof SA60AT | Incidence of Nd:YAG capsulotomy |
| Johansson 2017 | Sweden | -- | 50 patients (100 eyes) | 3 years | AcrySof IQ SN60WF vs. Tecnis ZCB00 | Incidence of Nd:YAG capsulotomy |
| Kahraman 2017 | Austria | October 2009 to February 2016 | 50 patients (100 eyes) | 5 years | Acrysof SA60AT vs. Tecnis ZCB00 | Incidence of Nd:YAG capsulotomy |
| Leydolt 2017 | Austria | June 2012 to December 2012 | 80 patients (160 eyes) | 3 years | Eyecee One vs. Acrysof SN60WF | Incidence of Nd:YAG capsulotomy |
| Kalauz 2016 | Croatia | January 2010 to December 2010 | 83 patients | 3 years | Acrysof SA60AT vs. Akreos Adapt AO | Incidence of Nd:YAG capsulotomy |
| Nagata 2015 | -- | -- | 60 eyes | 3 years | SP2 Hoya vs. FY-60AD Hoya | Incidence of Nd:YAG capsulotomy |
| Findl 2015 | UK, Austria | -- | 50 patients (100 eyes) | 2 years | Tecnis ZCB00 1-piece IOL vs. Tecnis ZA9003 3-piece IOL | Incidence of Nd:YAG capsulotomy |
| Kahraman 2015 | Austria | October 2009 to September 2013 | 80 patients (160 eyes) | 3 years | AcrySof SA60AT vs. Tecnis ZCB00 | Incidence of Nd:YAG capsulotomy |
| Krall 2015 | Austria | -- | 51 patients (102 eyes) | 12 months | EC-1Y PAL (uncoated IOL) vs. EC-1YH PAL or Polylens Y10 AS (HSM coated IOLs - no-subgroup data provided for these two) | Incidence of Nd:YAG capsulotomy |
| Schriefl 2015 (NCT01734343) | Austria | January to June 2009 | 65 patients (130 eyes) | 3 years | Y-60H vs. Micro AY | Incidence of Nd:YAG capsulotomy |
| Schriefl 2015 | Austria | March 2008 to January 2009 | 60 patients (120 eyes) | 4 years | Y-60H vs. Akreos MI60 | Incidence of Nd:YAG capsulotomy |
| Kahraman 2014 | Austria | October 2009 to September 2010 | 100 patients (200 eyes) | 12 months | AcrySof SA60AT vs. Tecnis ZCB00 | Incidence of Nd:YAG capsulotomy |
| Mylonas 2014 | Austria | -- | 62 patients (124 eyes) | 12 months | Corneal A501D IOL vs. Corneal J501D | Incidence of Nd:YAG capsulotomy |
| Ronbeck 2014 (Ronbeck 2009, Wegde 2004, Wegde 2003) | Sweden | May 1995 to March 1998 | 180 patients (180 eyes) Subgroup analysis (Wegde 2004): 119 patients | 12 years | CeeOn 809C vs. SI-40NB vs. Acrysof MA60BM | Incidence of Nd:YAG capsulotomy |
| Chang 2013 | Sweden | May 2003 to April 2005 | 80 patients | Mean 68 ± 6.8 months | Acrysof SA60AT vs. Sensar AR40e | Incidence of Nd:YAG capsulotomy |
| Hirnschall 2013 | UK | -- | 25 patients (50 eyes) | 3 years | Acri.Smart 46S (CT SPHERIS 209M) vs. AcriLyc 53N (CT 53N) | Incidence of Nd:YAG capsulotomy |
| Leydolt 2013 | Austria | April to June 2008 | 54 patients (108 eyes) | 3 years | Acrysof SA60AT vs. Tecnis ZCB00 | Incidence of Nd:YAG capsulotomy |
| Leydolt and Schriefl 2013 (NCT01732484) | Austria | August 2009 to May 2010 | 100 patients (200 eyes) | 3 years | AcrySof SN60WF vs. iMics NY-60 | Incidence of Nd:YAG capsulotomy |
| Mylonas 2013 (NCT 00673803) | Austria | January to July 2009 | 40 patients (80 eyes) | 1 year | Polylens Y10 vs. PolylensY30 | Incidence of Nd:YAG capsulotomy |
| Nanavaty 2013 | UK | January 2008 to November 2008 | 42 patients (84 eyes) | 24 months | Acri.Smart 36A vs. Akreos MI60 | Incidence of Nd:YAG capsulotomy |
| Prinz 2013 | Austria | May 2009 to August 2009 | 40 patients (80 eyes) | 1 year | AF-1 NY-60 vs. AF-1 iMics Y-60H | Incidence of Nd:YAG capsulotomy |
| Nanavaty 2012 (NCT00762021) | UK | November 2006 to July 2007 | 52 patients (104 eyes) | 24 months | AcrySof SA60AT vs. AcrySof SN60WF | Incidence of Nd:YAG capsulotomy |
| Gangwani 2011 | UK | February 2007 to July 2007 | 35 patients (70 eyes) | 24 months | Idea 613 XC vs. Acrysof SA60AT | Incidence of Nd:YAG capsulotomy |
| Iwase 2011 | Japan | January 2006 to August 2006 | 63 patients (126 eyes) | 2 years | Acrysof SA60AT vs. Meridian HP60M | Incidence of Nd:YAG capsulotomy |
| Vasavada 2011 | India | January 2006 to March 2007 | 76 patients (152 eyes) | 3 years | Acrysof IQ SN60WF vs.C-flex 570C/Akreos Adapt | Incidence of Nd:YAG capsulotomy |
| Zemaitiene 2011 | Lithuania | 3 years | 107 patients (123 eyes) | 3 years | AcrySof MA30BA vs. AcrySof SA30AL vs. CeeOn 911A | Incidence of Nd:YAG capsulotomy |
| Cleary 2009 | UK | February 2005 to February 2006 | 32 patients | 24 months | HumanOptics MC611MI vs. AcrySof MA60AC | Incidence of Nd:YAG capsulotomy |
| Vock 2009 | Austria | -- | 53 (106 eyes) | 6 years | CeeOn 911A vs. AcrySof MA60BM | Incidence of Nd:YAG capsulotomy |
| Hayashi 2008 | Japan | March 2003 to July 2003 | 45 patients (90 eyes) | 24 months | AcrySof IOL MA60AC vs. Sensar AR40e | Incidence of Nd:YAG capsulotomy |
| Kang 2008 | Korea | February 2004 through February 2006 | 100 patients (100 eyes) | 12 months | BioVue 3 HSM vs. Sensar AR40e | Incidence of Nd:YAG capsulotomy |
| Kohnen 2008 (Mester 2004) | Germany | -- | 288 patients (576 eyes) | 3 years | CeeOn Edge 911A vs. AcrySof MA60BM; CeeOn Edge 911A vs. PhacoFlex SI40NB | Incidence of Nd:YAG capsulotomy |
| Kugelberg 2008 (Kugelberg 2006) | Sweden | 2002 to 2004 | 120 patients (120 eyes) | 2 years | BL27 vs. AcrySof SA60AT | Incidence of Nd:YAG capsulotomy |
| Barisic 2007 | Croatia | -- | 60 patients(120 eyes) | 6 months | Acrysof Natural IOL (SN60AT) vs. AcrySof MA60BM | Incidence of Nd:YAG capsulotomy |
| Buehl 2007 (Linked: Buehl 2004) | Austria | June 2001 to February 2002 | 52 patients (104 eyes) | 3 years | Clariflex sillicone sharp edge IOL vs. Phacoflex sillicone round edge IOL | Incidence of Nd:YAG capsulotomy |
| Hancox 2007 | UK | -- | 30 patients (60 eyes) | 2 years | AcrySof MA30AC vs. 1CU | Incidence of Nd:YAG capsulotomy |
| Hayashi 2007 | Japan | March 2003 to October 2003 | 100 patients (200 eyes) | 36 months | Sensar AR40e vs. ClariFlex | Incidence of Nd:YAG capsulotomy |
| Kaya 2007 | Turkey | -- | 25 patients (50 eyes) | Mean 12.8±1.5 months (range 11 to 14 months) | Ultrachoice 1.0 rollable ThinOptx vs. AcrySof MA30AC | Incidence of Nd:YAG capsulotomy |
| Leydolt 2007 | Austria | October 2000 to June 2001 | 52 patients (104 eyes) | 5 years | AcrySof SA30AL vs. AcrySof MA30BA or AcrySof SA60AT vs. Acrysof MA60BM | Incidence of Nd:YAG capsulotomy |
| Vock 2007 | Austria | -- | 52 patients (104 eyes) | 1.5 years | ACR6D SE vs. C-flex 570C | Incidence of Nd:YAG capsulotomy |
| Zemaitiene 2007 | Lithuania | -- | 74 patients (74 eyes) | 2 years | AcrySof MA30BA vs. AcrySof SA30AL | Incidence of Nd:YAG capsulotomy |
| Buehl 2002 (Linked: Buehl 2005) | Austria | December 1999 to June 2000 | 53 patients (106 eyes) | 3 years | Sensar AR40e vs. Sensar AR40 | Incidence of Nd:YAG capsulotomy |
| Findl 2005 | Austria | -- | 53 patients (106 eyes) | 3 years | Acrysof MA60BM vs. CeeOn 911A | Incidence of Nd:YAG capsulotomy |
| Hayashi 2005 | Japan | October 2001 to March 2002 | 75 patients (150 eyes) | 24 months | Sensar AR40e vs. Sensar AR40 | Incidence of Nd:YAG capsulotomy |
| Heatly 2005 | UK | -- | 53 patients (106 eyes) | 1 year | Centerflex 570H IOL vs. AcrySof SA60AT IOL IOL | Incidence of Nd:YAG capsulotomy |
| Menapace 2005 | Austria | April 1999 to February 2000 | 54 patients (108 eyes) | 3 years | Allergan SI40NB vs. Domilens Silens6 | Incidence of Nd:YAG capsulotomy |
| Sacu 2005 | Germany | November 1998 to June 1999 | 51 patients (102 eyes) | Mean 62.5 ± 3.1 months | Microsil Model S vs. Microsil Model R | Incidence of Nd:YAG capsulotomy |
| Sundelin-2005 (Linked: Sundelin-2006) | Sweden | 2000 to 2001 | 116 patients | 5 years | CeeOn 911A vs. SI40NB | Incidence of Nd:YAG capsulotomy |
| Hayashi 2004 | Japan | December 1999 to June 2002 | 100 patients (200 eyes) | 24 months | Hydroview H60M vs. AcrySof MA60BM | Incidence of Nd:YAG capsulotomy |
| Sacu 2004 | Austria | October 2000 to July 2001 | 52 patients (104 eyes) | 2 years | Acrysof SA30AL or SA60AT vs. Acrysof MA30BA or MA60BM | Incidence of Nd:YAG capsulotomy |
| Sacu 2004 | Austria | -- | 105 patients (210 eyes) | 1 year | Group1: Sensar AR40e vs. AR40  Group 2: ClariFlex OptiEdge vs. SI40NB | Incidence of Nd:YAG capsulotomy |
| Sacu 2004 | Austria | -- | 72 patients (144 eyes) | 1 year | Sensar AR40 vs. Sensar AR40e vs. CeeOn 911A | Incidence of Nd:YAG capsulotomy |
| Ernest 2003 | US | -- | 156 patients | Mean 3 years | AcrySof MA30BA vs. PhacoFlex II SI-40NB | Incidence of Nd:YAG capsulotomy |
| Hayashi 2001 | Japan | May 1997 to September 1997 | 300 patients (300 eyes) | 24 months | MZ60BD vs. SI30NB vs. Acrysof MA60BM | Incidence of Nd:YAG capsulotomy |
| Hollick 2000 | -- | February 1995 to September 1996 | 93 patients (93 eyes) | 2 years | Storz P497UV vs. SI30 vs. Hydroview H60M | Incidence of Nd:YAG capsulotomy |
| Kobayashi 2000 | -- | January 1995 to May 1998 | Total: 2192 patients (2778 eyes) 1 year follow-up: 1202 patients (1514 eyes) 3 year follow-up: 990 patients (1264 eyes) | 1 year, 3 year | AcrySof MA60BM vs. AcrySof MC60BM | Incidence of Nd:YAG capsulotomy |
| Kucuksumer 2000 | Turkey | -- | 50 patients (100 eyes) | 3 years | AcrySof MA60BM vs. Memory lens U940A | Incidence of Nd:YAG capsulotomy |
| Wang-2000 | Taiwan, Republic of China. | March to April 1997 | 40 eyes | 1 year | CeeOn 812A vs. SI30NB | Incidence of Nd:YAG capsulotomy |
| Hollick 1999 | Britain | September 1993 to July 1994 | 81 patients (90 eyes) | 3 years | MC60BM vs. Iolab LI41U vs. AcrySof MA60BM | Incidence of Nd:YAG capsulotomy |
| Hayashi 1998 | -- | -- | 240 patients (240 eyes) | 2 years | MZ60BD vs. SI30NB vs. Acrysof MA60BM | Incidence of Nd:YAG capsulotomy |
| Ursell 1998 | Britain | September 1993 to July 1994 | 81 patients (90 eyes) | 2 years | MC60BM vs. Iolab LI41 U vs. AcrySof MA60BM | Incidence of Nd:YAG capsulotomy |
| Hollick 2000 | -- | February 1995 to September 1996 | 93 patients (93 eyes) | 2 years | Storz P497UV vs. SI30 vs. Hydroview H60M | Incidence of Nd:YAG capsulotomy |
| Kobayashi 2000 | -- | January 1995 to May 1998 | Total: 2192 patients (2778 eyes) 1 year follow-up: 1202 patients (1514 eyes) 3 year follow-up: 990 patients (1264 eyes) | 1 year, 3 year | AcrySof MA60BM vs. AcrySof MC60BM | Incidence of Nd:YAG capsulotomy |
| Kucuksumer 2000 | Turkey | -- | 50 patients (100 eyes) | 3 years | AcrySof MA60BM vs. Memory lens U940A | Incidence of Nd:YAG capsulotomy |
| Wang-2000 | Taiwan, Republic of China. | March to April 1997 | 40 eyes | 1 year | CeeOn 812A vs. SI30NB | Incidence of Nd:YAG capsulotomy |
| Hollick 1999 | Britain | September 1993 to July 1994 | 81 patients (90 eyes) | 3 years | MC60BM vs. Iolab LI41U vs. AcrySof MA60BM | Incidence of Nd:YAG capsulotomy |
| Hayashi 1998 | -- | -- | 240 patients (240 eyes) | 2 years | MZ60BD vs. SI30NB vs. Acrysof MA60BM | Incidence of Nd:YAG capsulotomy |
| Ursell 1998 | Britain | September 1993 to July 1994 | 81 patients (90 eyes) | 2 years | MC60BM vs. Iolab LI41 U vs. AcrySof MA60BM | Incidence of Nd:YAG capsulotomy |

**Citations**

CHANG, A. & KUGELBERG, M. 2017. Posterior capsule opacification 9 years after phacoemulsification with a hydrophobic and a hydrophilic intraocular lens. European Journal of Ophthalmology, 27, 164-168.

HARIPRIYA, A., CHANG, D. F., VIJAYAKUMAR, B., NIRAJ, A., SHEKHAR, M., TANPREET, S. & ARAVIND, S. 2017. Long-term Posterior Capsule Opacification Reduction with Square-Edge Polymethylmethacrylate Intraocular Lens: Randomized Controlled Study. Ophthalmology, 124, 295-302.

JOHANSSON, B. 2017. Glistenings, anterior/posterior capsular opacification and Incidence of Nd:YAG laser treatments with two aspheric hydrophobic acrylic intraocular lenses - a long-term intra-individual study. Acta Ophthalmologica.

KAHRAMAN, G., FERDINARO, C., WETZEL, B., BERNHART, C., PRAGER, F. & AMON, M. 2017. Intraindividual comparison of capsule behavior of 2 hydrophobic acrylic intraocular lenses during a 5-year follow-up. Journal of Cataract and Refractive Surgery, 43, 228-233.

LEYDOLT, C., SCHARTMÜLLER, D., SCHWARZENBACHER, L., SCHRANZ, M., SCHRIEFL, S. & MENAPACE, R. 2017. Comparison of posterior capsule opacification development with 2 single-piece intraocular lens types. Journal of Cataract and Refractive Surgery, 43, 774-780.

KALAUZ, M., MASNEC, S., KORDIĆ, R., KUZMAN, T., VIDAS, S., ŠKEGRO, I., JANDROKOVIĆ, S. & PERIĆ, S. 2016. Posterior Capsule Opacification and Nd:YAG Rates with Two Acrylic Intraocular Lenses after Age-Related Cataract Treatment: Three-year Results. Seminars in Ophthalmology, 1-7.

NAGATA, M., MATSUSHIMA, H., MUKAI, K., GOTOH, N., TERAUCHI, W. & SENOO, T. 2015. Postoperative outcomes using surface modified intraocular lens. Investigative Ophthalmology and Visual Science, 56, 653.

FINDL, O., HIRNSCHALL, N., NISHI, Y., MAURINO, V. & CRNEJ, A. 2015. Capsular bag performance of a hydrophobic acrylic 1-piece intraocular lens. Journal of Cataract and Refractive Surgery, 41, 90-97.

KAHRAMAN, G., AMON, M., FERDINARO, C., NIGL, K. & WALCH, M. 2015. Intraindividual comparative analysis of capsule opacification after implantation of 2 single-piece hydrophobic acrylic intraocular lenses models: Three-year follow-up. Journal of Cataract and Refractive Surgery, 41, 990-996.

KRALL, E. M., ARLT, E. M., JELL, G., STROHMAIER, C., MOUSSA, S. & DEXL, A. K. 2015. Prospective randomized intraindividual comparison of posterior capsule opacification after implantation of an IOL with and without heparin surface modification. Journal of Refractive Surgery, 31, 466-472.

SCHRIEFL, S. M., LEYDOLT, C., STIFTER, E. & MENAPACE, R. 2015. Posterior capsular opacification and Nd:YAG capsulotomy rates with the iMics Y-60H and Micro AY intra-ocular lenses: 3-year results of a randomized clinical trial. Acta Ophthalmologica, 93, 342-347.

SCHRIEFL, S. M., MENAPACE, R., STIFTER, E., ZARUBA, D. & LEYDOLT, C. 2015b. Posterior capsule opacification and neodymium:YAG laser capsulotomy rates with 2 microincision intraocular lenses: Four-year results. Journal of Cataract and Refractive Surgery, 41, 956-963.

HENNIG, A., PURI, L. R., SHARMA, H., EVANS, J. R. & YORSTON, D. 2014. Foldable vs rigid lenses after phacoemulsification for cataract surgery: A randomised controlled trial. Eye (Basingstoke), 28, 567-575.

KAHRAMAN, G., SCHRITTWIESER, H., WALCH, M., STORCH, F., NIGL, K., FERDINARO, C. & AMON, M. 2014. Anterior and posterior capsular opacification with the Tecnis ZCB00 and AcrySof SA60AT IOLs: A randomised intraindividual comparison. British Journal of Ophthalmology, 98, 905-909.

MYLONAS, G., GEORGOPOULOS, M., PRINZ, A., VOCK, L., BLUM, R. A. & SCHMIDT-ERFURTH, U. 2014. Influence of a variable overall diameter hydrophilic acrylic sharp-edged single-piece intra-ocular lens on capsule opacification one year after surgery. Current Eye Research, 39, 620-625.

"RØNBECK, M. & KUGELBERG, M. 2014. Posterior capsule opacification with 3 intraocular lenses: 12-year prospective study. Journal of Cataract and Refractive Surgery, 40, 70-76.

RÖNBECK, M., ZETTERSTRÖM, C., WEJDE, G. & KUGELBERG, M. 2009. Comparison of posterior capsule opacification development with 3 intraocular lens types. Five-year prospective study. Journal of Cataract and Refractive Surgery, 35, 1935-1940.

WEJDE, G., KUGELBERG, M. & ZETTERSTRÖM, C. 2004. Position of anterior capsulorhexis and posterior capsule opacification. Acta Ophthalmologica Scandinavica, 82, 531-534.

WEJDE, G., KUGELBERG, M. & ZETTERSTRÖM, C. 2003. Posterior capsule opacification: Comparison of 3 intraocular lenses of different materials and design. Journal of Cataract and Refractive Surgery, 29, 1556-1559."

CHANG, A., BEHNDIG, A., RØNBECK, M. & KUGELBERG, M. 2013. Comparison of posterior capsule opacification and glistenings with 2 hydrophobic acrylic intraocular lenses: 5- to 7-year follow-up. Journal of Cataract and Refractive Surgery, 39, 694-698.

HIRNSCHALL, N., NISHI, Y., CRNEJ, A., KOSHY, J., GANGWANI, V., MAURINO, V. & FINDL, O. 2013. Capsular bag stability and posterior capsule opacification of a plate-haptic design microincision cataract surgery intraocular lens: 3-Year results of a randomised trial. British Journal of Ophthalmology, 97, 1565-1568.

LEYDOLT, C., KRIECHBAUM, K., SCHRIEFL, S., PACHALA, M. & MENAPACE, R. 2013a. Posterior capsule opacification and neodymium:YAG rates with 2 single-piece hydrophobic acrylic intraocular lenses: Three-year results. Journal of Cataract and Refractive Surgery, 39, 1886-1892.

LEYDOLT, C., SCHRIEFL, S., STIFTER, E., HASZCZ, A. & MENAPACE, R. 2013b. Posterior capsule opacification with the iMics1 NY-60 and AcrySof SN60WF 1-piece hydrophobic acrylic intraocular lenses: 3-year results of a randomized trial. American Journal of Ophthalmology, 156, 375-381.e2.

MYLONAS, G., PRSKAVEC, M., BARADARAN-DILMAGHANI, R., KARNIK, N., BUEHL, W. & WIRTITSCH, M. 2013. Effect of a single-piece and a three-piece acrylic sharp-edged IOL on posterior capsule opacification. Current Eye Research, 38, 86-90.

NANAVATY, M. A., SPALTON, D. J., GALA, K. B., DHITAL, A. & BOYCE, J. 2013. Fellow-eye comparison of posterior capsule opacification between 2 aspheric microincision intraocular lenses. Journal of Cataract and Refractive Surgery, 39, 705-711.

PRINZ, A., VECSEI-MARLOVITS, P. V., SONDERHOF, D., IRSIGLER, P., FINDL, O. & WEINGESSEL, B. 2013. Comparison of posterior capsule opacification between a 1-piece and a 3-piece microincision intraocular lens. British Journal of Ophthalmology, 97, 18-22.

NANAVATY, M. A., SPALTON, D. J., GALA, K. B., DHITAL, A. & BOYCE, J. 2012. Effect of intraocular lens asphericity on posterior capsule opacification between two intraocular lenses with same acrylic material: A fellow-eye study. Acta Ophthalmologica, 90, e104-e108.

GANGWANI, V., HIRNSCHALL, N., KOSHY, J., CRNEJ, A., NISHI, Y., MAURINO, V. & FINDL, O. 2011. Posterior capsule opacification and capsular bag performance of a microincision intraocular lens. Journal of Cataract and Refractive Surgery, 37, 1988-1992.

IWASE, T., NISHI, Y., OVESON, B. C. & JO, Y. J. 2011. Hydrophobic versus double-square-edged hydrophilic foldable acrylic intraocular lens: Effect on posterior capsule opacification. Journal of Cataract and Refractive Surgery, 37, 1060-1068.

PRINZ, A., NEUMAYER, T., BUEHL, W., VOCK, L., MENAPACE, R., FINDL, O. & GEORGOPOULOS, M. 2011. Rotational stability and posterior capsule opacification of a plate-haptic and an open-loop-haptic intraocular lens. Journal of Cataract and Refractive Surgery, 37, 251-257.

VASAVADA, A. R., RAJ, S. M., SHAH, A., SHAH, G., VASAVADA, V. & VASAVADA, V. 2011. Comparison of posterior capsule opacification with hydrophobic acrylic and hydrophilic acrylic intraocular lenses. Journal of Cataract and Refractive Surgery, 37, 1050-1059.

ŽEMAITIENE, R. & JAŠINSKAS, V. 2011. Prevention of posterior capsule opacification with 3 intraocular lens models: A prospective, randomized, long-term clinical trial. Medicina, 47, 595-599.

CLEARY, G., SPALTON, D. J., HANCOX, J., BOYCE, J. & MARSHALL, J. 2009. Randomized intraindividual comparison of posterior capsule opacification between a microincision intraocular lens and a conventional intraocular lens. Journal of Cataract and Refractive Surgery, 35, 265-272.

VOCK, L., CRNEJ, A., FINDL, O., NEUMAYER, T., BUEHL, W., SACU, S., RAINER, G. & MENAPACE, R. 2009. Posterior Capsule Opacification in Silicone and Hydrophobic Acrylic Intraocular Lenses with Sharp-edge Optics Six Years After Surgery. American Journal of Ophthalmology, 147, 683-690.e2.

HANCOX, J., SPALTON, D., CLEARY, G., BOYCE, J., NANAVATY, M. A., THYAGARAJAN, S. & MARSHALL, J. 2008. Fellow-eye comparison of posterior capsule opacification with AcrySof SN60AT and AF-1 YA-60BB blue-blocking intraocular lenses. Journal of Cataract and Refractive Surgery, 34, 1489-1494.

HAYASHI, K., YOSHIDA, M. & HAYASHI, H. 2008. Comparison of posterior capsule opacification between fellow eyes with two types of acrylic intraocular lens. Eye, 22, 35-41.

KANG, S., KIM, M. J., PARK, S. H. & JOO, C. K. 2008. Comparison of clinical results between heparin surface modified hydrophilic acrylic and hydrophobic acrylic intraocular lens. European Journal of Ophthalmology, 18, 377-383.

KHANZADA, M. A., GUL, S., DABIR, S. A., JATOI, S. M. & NARSANI, A. K. 2008. Comparative Incidence of posterior capsular opacification in AcrySof and PMMA intraocular lenses. International Journal of Ophthalmology, 8, 2369-2372.

"KOHNEN, T., FABIAN, E., GERL, R., HUNOLD, W., HÜTZ, W., STROBEL, J., HOYER, H. & MESTER, U. 2008. Optic Edge Design as Long-term Factor for Posterior Capsular Opacification Rates. Ophthalmology, 115, 1308-1314.e3.

MESTER, U., FABIAN, E., GERL, R., HUNOLD, W., HÜTZ, W., STROBEL, J., HOYER, H. & KOHNEN, T. 2004. Posterior capsule opacification after implantation of CeeOn Edge 911A, PhacoFlex SI-40NB, and AcrySof MA60BM lenses: One-year results of an intraindividual comparison multicenter study. Journal of Cataract and Refractive Surgery, 30, 978-985."

"KUGELBERG, M., WEJDE, G., JAYARAM, H. & ZETTERSTRÖM, C. 2008. Two-year follow-up of posterior capsule opacification after implantation of a hydrophilic or hydrophobic acrylic intraocular lens. Acta Ophthalmologica, 86, 533-536.

KUGELBERG, M., WEJDE, G., JAYARAM, H. & ZETTERSTRÖM, C. 2006. Posterior capsule opacification after implantation of a hydrophilic or a hydrophobic acrylic intraocular lens. One-year follow-up. Journal of Cataract and Refractive Surgery, 32, 1627-1631."

SHAH, A. R., PRAVEEN, M. R. & VASAVADA, A. R. 2008. Posterior capsule opacification after extra capsular cataract extraction in Indian rural population: Foldable acrylic vs poly (methyl-methacrylate) intraocular lenses a randomized clinical trial. Eye, 22, 889-894.

BARIŠIĆ, A., DEKARIS, I., GABRIĆ, N., BOSNAR, D., LAZIĆ, R., MARTINOVIĆ, Ž. K. & KRSTONIJEVIĆ, E. K. 2007. Blue light filtering intraocular lenses in phacoemulsification cataract surgery. Collegium Antropologicum, 31, 57-60.

"BUEHL, W., MENAPACE, R., FINDL, O., NEUMAYER, T., BOLZ, M. & PRINZ, A. 2007. Long-term Effect of Optic Edge Design in a Silicone Intraocular Lens on Posterior Capsule Opacification. American Journal of Ophthalmology, 143, 913-919.e2.

BUEHL, W., MENAPACE, R., SACU, S., KRIECHBAUM, K., KOEPPL, C., WIRTITSCH, M., GEORGOPOULOS, M. & FINDL, O. 2004. Effect of a silicone intraocular lens with a sharp posterior optic edge on posterior capsule opacification. Journal of Cataract and Refractive Surgery, 30, 1661-1667."

HANCOX, J., SPALTON, D., HEATLEY, C., JAYARAM, H., YIP, J., BOYCE, J. & MARSHALL, J. 2007. Fellow-eye comparison of posterior capsule opacification rates after implantation of 1CU accommodating and AcrySof MA30 monofocal intraocular lenses. Journal of Cataract and Refractive Surgery, 33, 413-417.

HAYASHI, K. & HAYASHI, H. 2007. Influence on Posterior Capsule Opacification and Visual Function of Intraocular Lens Optic Material. American Journal of Ophthalmology, 144, 195-202.e2.

KAYA, V., ÖZTÜRKER, Z. K., ÖZTÜRKER, C., YAŞAR, Ö., SIVRIKAYA, H., AǦCA, A. & YILMAZ, Ö. F. 2007. ThinOptX vs AcrySof: Comparison of visual and refractive results, contrast sensitivity, and the Incidence of posterior capsule opacification. European Journal of Ophthalmology, 17, 307-314.

LEYDOLT, C., DAVIDOVIC, S., SACU, S., MENAPACE, R., NEUMAYER, T., PRINZ, A., BUEHL, W. & FINDL, O. 2007. Long-term Effect of 1-Piece and 3-Piece Hydrophobic Acrylic Intraocular Lens on Posterior Capsule Opacification. A Randomized Trial. Ophthalmology, 114, 1663-1669.

"VOCK, L., MENAPACE, R., STIFTER, E., FINDL, O. & GEORGOPOULOS, M. 2007. Clinical effects of primary posterior continuous curvilinear capsulorhexis in eyes with single-piece hydrophilic acrylic intraocular lenses with and without haptic angulation. J Cataract Refract Surg, 33, 258-64."

ZEMAITIENE, R., JASINSKAS, V. & AUFFARTH, G. U. 2007. Influence of three-piece and single-piece designs of two sharp-edge optic hydrophobic acrylic intraocular lenses on the prevention of posterior capsule opacification: A prospective, randomised, long-term clinical trial. British Journal of Ophthalmology, 91, 644-648.

"BUEHL, W., FINDL, O., MENAPACE, R., RAINER, G., SACU, S., KISS, B., PETTERNEL, V. & GEORGOPOULOS, M. 2002. Effect of an acrylic intraocular lens with a sharp posterior optic edge on posterior capsule opacification. Journal of Cataract and Refractive Surgery, 28, 1105-1111.

BUEHL, W., FINDL, O., MENAPACE, R., SACU, S., KRIECHBAUM, K., KOEPPL, C. & WIRTITSCH, M. 2005. Long-term effect of optic edge design in an acrylic intraocular lens on posterior capsule opacification. Journal of Cataract and Refractive Surgery, 31, 954-961."

FINDL, O., MENAPACE, R., SACU, S., BUEHL, W. & RAINER, G. 2005. Effect of optic material on posterior capsule opacification in intraocular lenses with sharp-edge optics: Randomized clinical trial. Ophthalmology, 112, 67-72.

HAYASHI, K. & HAYASHI, H. 2005. Posterior capsule opacification in the presence of an intraocular lens with a sharp versus rounded optic edge. Ophthalmology, 112, 1550-1556.

HEATLEY, C. J., SPALTON, D. J., KUMAR, A., JOSE, R., BOYCE, J. & BENDER, L. E. 2005. Comparison of posterior capsule opacification rates between hydrophilic and hydrophobic single-piece acrylic intraocular lenses. J Cataract Refract Surg, 31, 718-24.

MENAPACE, R., WIRTITSCH, M., FINDL, O., BUEHL, W., KRIECHBAUM, K. & SACU, S. 2005. Effect of anterior capsule polishing on posterior capsule opacification and neodymium:YAG capsulotomy rates: Three-year randomized trial. Journal of Cataract and Refractive Surgery, 31, 2067-2075.

SACU, S., MENAPACE, R., FINDL, O., KISS, B., BUEHL, W. & GEORGOPOULOS, M. 2005. Long-term efficacy of adding a sharp posterior optic edge to a three-piece silicone intraocular lens on capsule opacification: five-year results of a randomized study. American journal of ophthalmology, 139, 696-703.

"SUNDELIN, K., SHAMS, H. & STENEVI, U. 2005. Three-year follow-up of posterior capsule opacification with two different silicone intraocular lenses. Acta Ophthalmologica Scandinavica, 83, 11-19.

SUNDELIN, K. & STENEVI, U. 2006. Five-year follow-up of posterior capsule opacification with two different silicone intraocular lenses. Acta Ophthalmologica Scandinavica, 84, 827-828."

BILGE, A. H., AYKAN, Ü., AKIN, T. & ÜNSAL, U. 2004. The effects of three-piece or single-piece acrylic intraocular lens implantation on posterior capsule opacification. European Journal of Ophthalmology, 14, 375-380.

HAYASHI, K. & HAYASHI, H. 2004. Posterior capsule opacification after implantation of a hydrogel intraocular lens. British Journal of Ophthalmology, 88, 182-185.

SACU, S., FINDL, O., MENAPACE, R., BUEHL, W. & WIRTITSCH, M. 2004. Comparison of posterior capsule opacification between the 1-piece and 3-piece Acrysof intraocular lenses: Two-year results of a randomized trial. Ophthalmology, 111, 1840-1846.

SACU, S., MENAPACE, R., BUEHL, W., RAINER, G. & FINDL, O. 2004. Effect of intraocular lens optic edge design and material on fibrotic capsule opacification and capsulorhexis contraction. Journal of Cataract and Refractive Surgery, 30, 1875-1882.

SACU, S., MENAPACE, R., FINDL, O., GEORGOPOULOS, M., BUEHL, W., KRIECHBAUM, K. & RAINER, G. 2004. Influence of optic edge design and anterior capsule polishing on posterior capsule fibrosis. Journal of Cataract and Refractive Surgery, 30, 658-662.

ERNEST, P. H. 2003. Posterior capsule opacification and neodymium: YAG capsulotomy rates with AcrySof acrylic and PhacoFlex II silicone intraocular lenses. Journal of Cataract and Refractive Surgery, 29, 1546-1550.

ABELA-FORMANEK, C., AMON, M., SCHILD, G., SCHAUERSBERGER, J., HEINZE, G. & KRUGER, A. 2002. Uveal and capsular biocompatibility of hydrophilic acrylic, hydrophobic acrylic, and silicone intraocular lenses. Journal of Cataract and Refractive Surgery, 28, 50-61.

HAYASHI K, H. H. N. F. H. F. 2001. Changes in posterior capsule opacification after poly(methyl methacrylate), silicone, and acrylic intraocular lens implantation. Journal of cataract and refractive surgery, 27, 817.

HOLLICK, E. J., SPALTON, D. J., URSELL, P. G., MEACOCK, W. R., BARMAN, S. A. & BOYCE, J. F. 2000. Posterior capsular opacification with hydrogel, polymethylmethacrylate, and silicone intraocular lenses: two-year results of a randomized prospective trial. American Journal of Ophthalmology, 129, 577-584.

KOBAYASHI, H., IKEDA, H., IMAMURA, S., KOBAYASHI, K., MITSUMA, Y., MINAMI, M., ISHIDA, O., HIROSE, M. & KOBAYASHI, K. 2000. Clinical assessment of long-term safety and efficacy of a widely implanted polyacrylic intraocular lens material. American Journal of Ophthalmology, 130, 310-321.

KÜÇÜKSÜMER, Y., BAYRAKTAR, Ş., ŞAHIN, Ş. & YILMAZ, Ö. F. 2000. Posterior capsule opacification 3 years after implantation of an AcrySof and a MemoryLens in fellow eyes. Journal of Cataract and Refractive Surgery, 26, 1176-1182.

WANG, M. C. & WOUNG, L. C. 2000. Digital retroilluminated photography to analyze posterior capsule opacification in eyes with intraocular lenses. Journal of Cataract and Refractive Surgery, 26, 56-61.

HOLLICK, E. J., SPALTON, D. J., URSELL, P. G., PANDE, M. V., BARMAN, S. A., BOYCE, J. F. & TILLING, K. 1999. The effect of polymethylmethacrylate, silicone, and polyacrylic intraocular lenses on posterior capsular opacification 3 years after cataract surgery. Ophthalmology, 106, 49-54; discussion 54-55.

HAYASHI, H., HAYASHI, K., NAKAO, F. & HAYASHI, F. 1998. Quantitative comparison of posterior capsule opacification after polymethylmethacrylate, silicone, and soft acrylic intraocular lens implantation. Archives of Ophthalmology, 116, 1579-1582.

URSELL, P. G., SPALTON, D. J., PANDE, M. V., HOLLICK, E. J., BARMAN, S., BOYCE, J. & TILLING, K. 1998. Relationship between intraocular lens biomaterials and posterior capsule opacification. Journal of Cataract and Refractive Surgery, 24, 352-360.
