# Supplementary material for: Effect of AcrySof versus other intraocular lens properties on the risk of Nd:YAG capsulotomy after cataract surgery: A systematic literature review and network meta-analysis
Source: PLoS One. 2019 Aug 19;14(8):e0220498. doi: 10.1371/journal.pone.0220498 (PMC6699683; doi:10.1371/journal.pone.0220498)
Supplement: S3 File — (ZIP) [file pone.0220498.s003.zip › S3_Files/S3_Additional details on Publication bias assessment.docx]

**S3 Supporting information**

**Additional details on assessment of risk of bias**

**S3 Fig. 1 - Funnel plots of the overall analysis for publication bias**


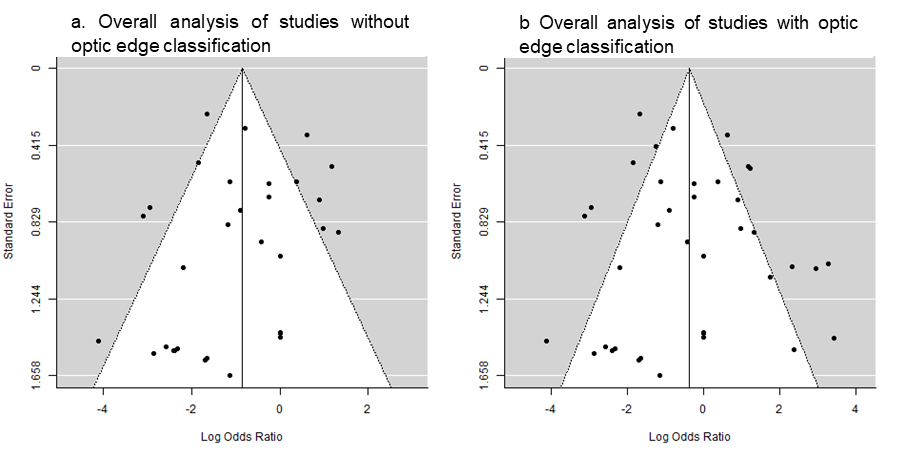


**S3 Fig. 2 - Assessing publication bias - Studies with AcrySof^®^ IOLs, other hydrophobic acrylic, hydrophilic acrylic, silicone, and PMMA IOLs without optic edge classification**

**
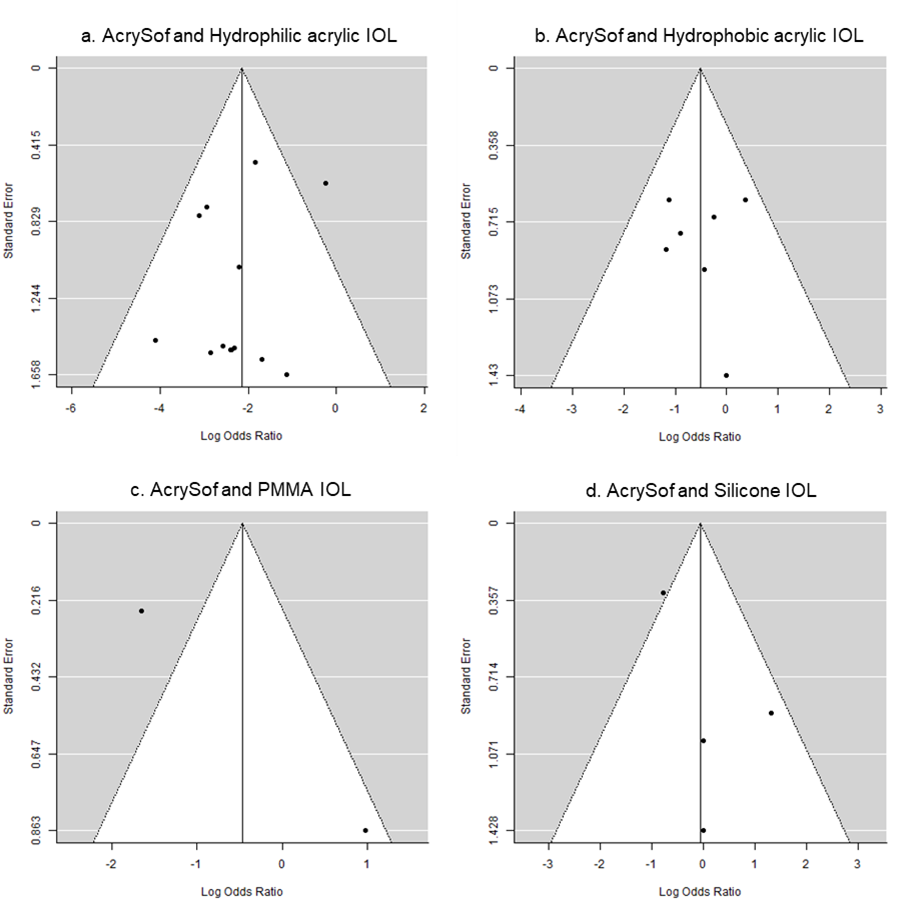
**

**
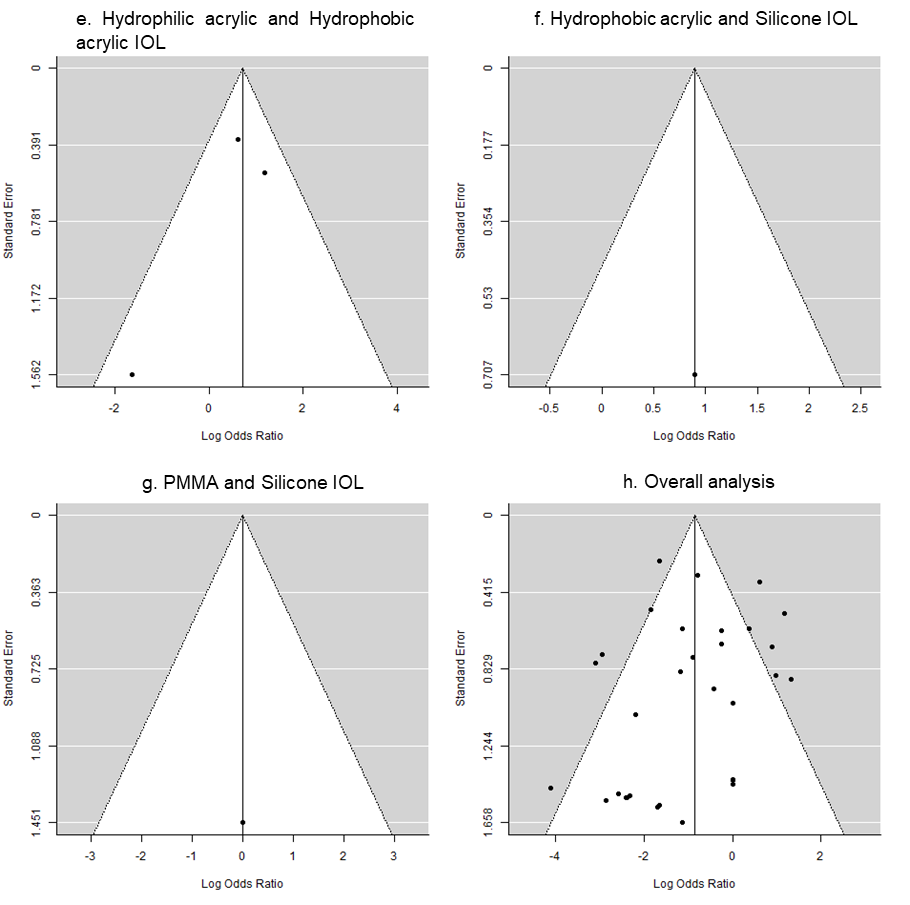
**

**S3 Fig. 3 - Assessing publication bias - Studies with AcrySof^®^ IOLs, other hydrophobic acrylic, hydrophilic acrylic, silicone, and PMMA IOLs with optic edge classification**

**
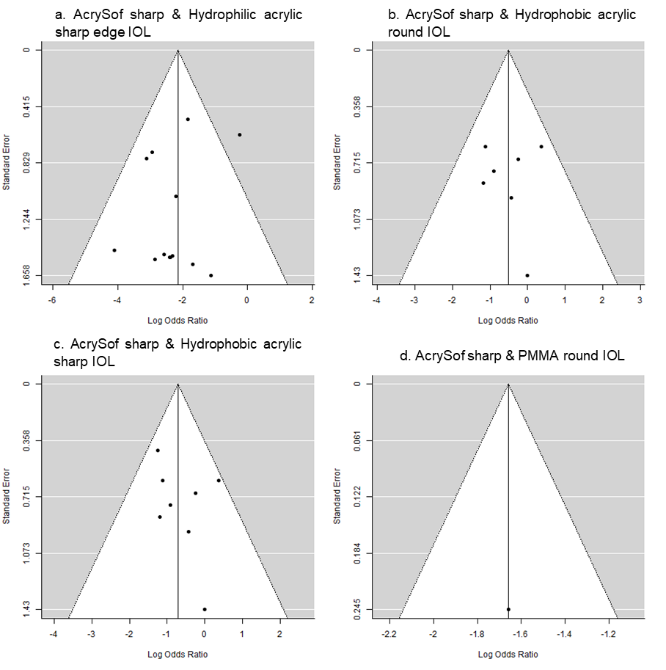
**

**
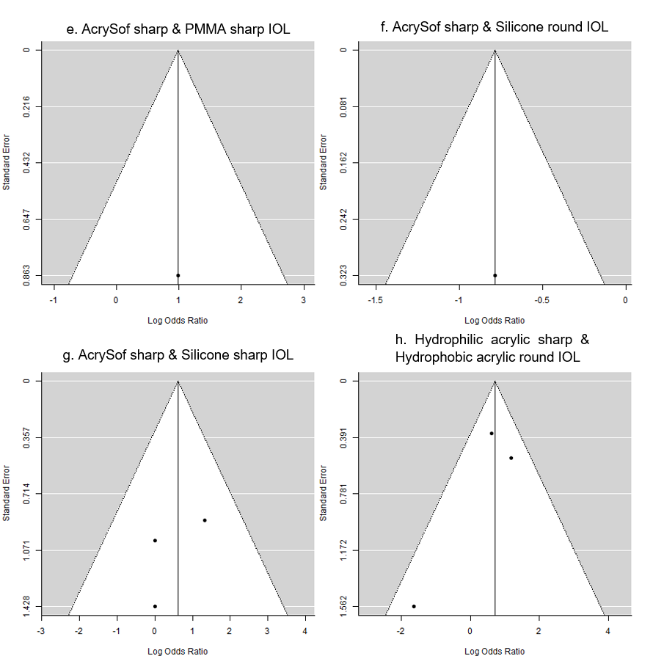
**

**
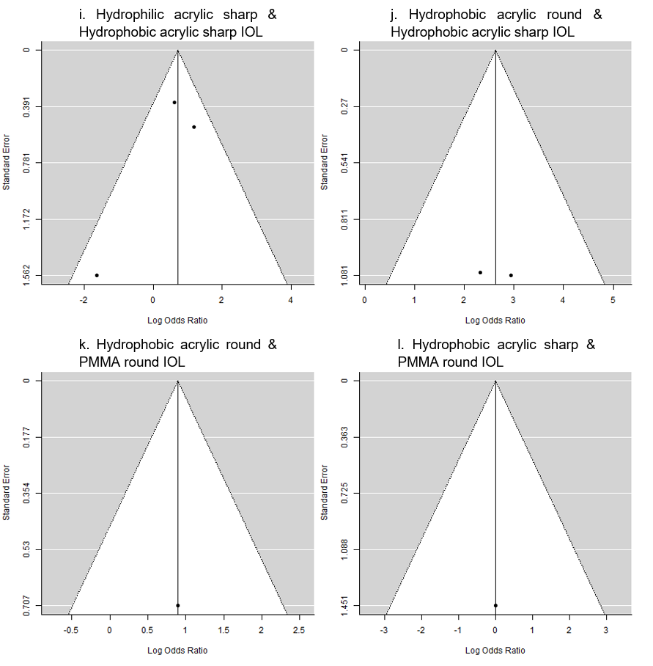
**

**
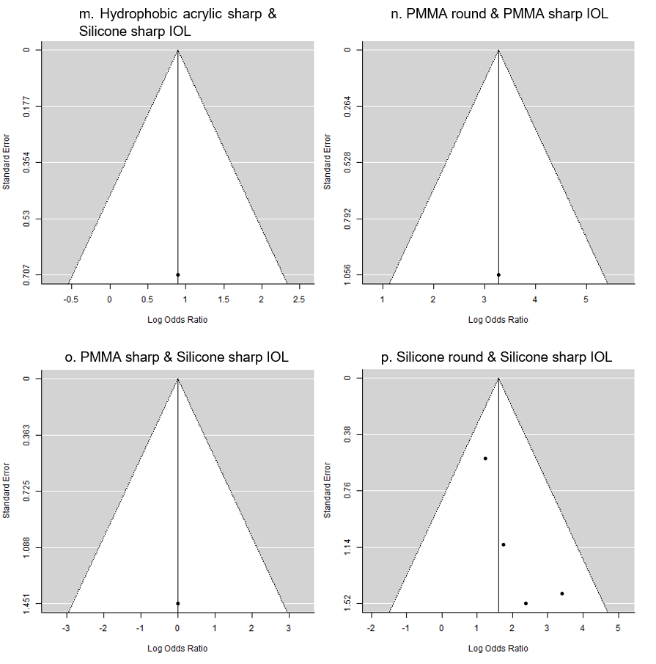
**
