# Supplementary figures and images for: Effect of AcrySof versus other intraocular lens properties on the risk of Nd:YAG capsulotomy after cataract surgery: A systematic literature review and network meta-analysis
Source: PLoS One. 2019 Aug 19;14(8):e0220498. doi: 10.1371/journal.pone.0220498 (PMC6699683; doi:10.1371/journal.pone.0220498)

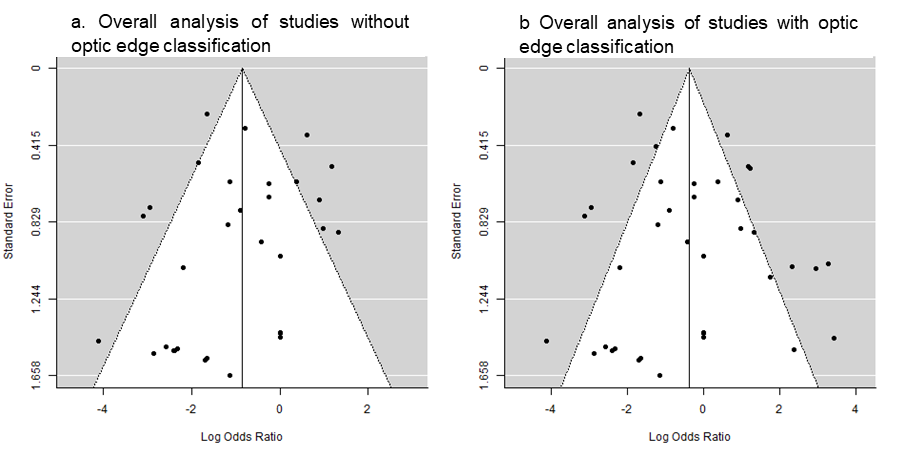

Supplement: S3 File — (ZIP) [file pone.0220498.s003.zip › S3_Files/S3_Fig-1.tif]

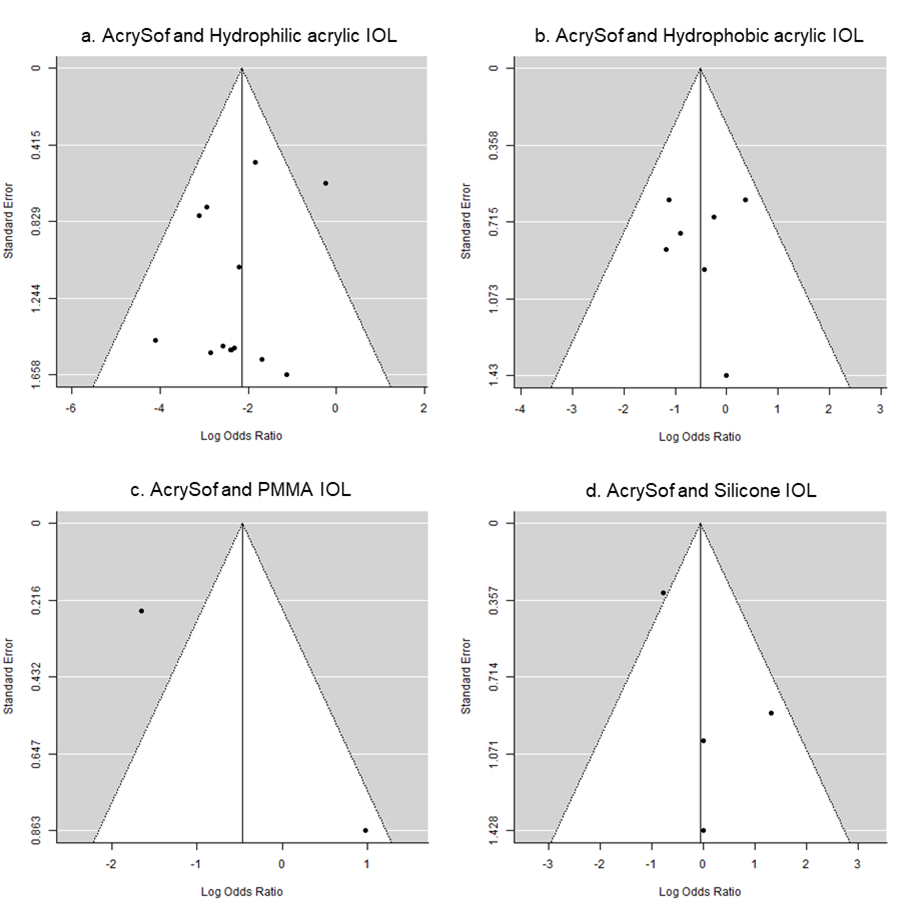

Supplement: S3 File — (ZIP) [file pone.0220498.s003.zip › S3_Files/S3_Fig-2 (a-d).tif]

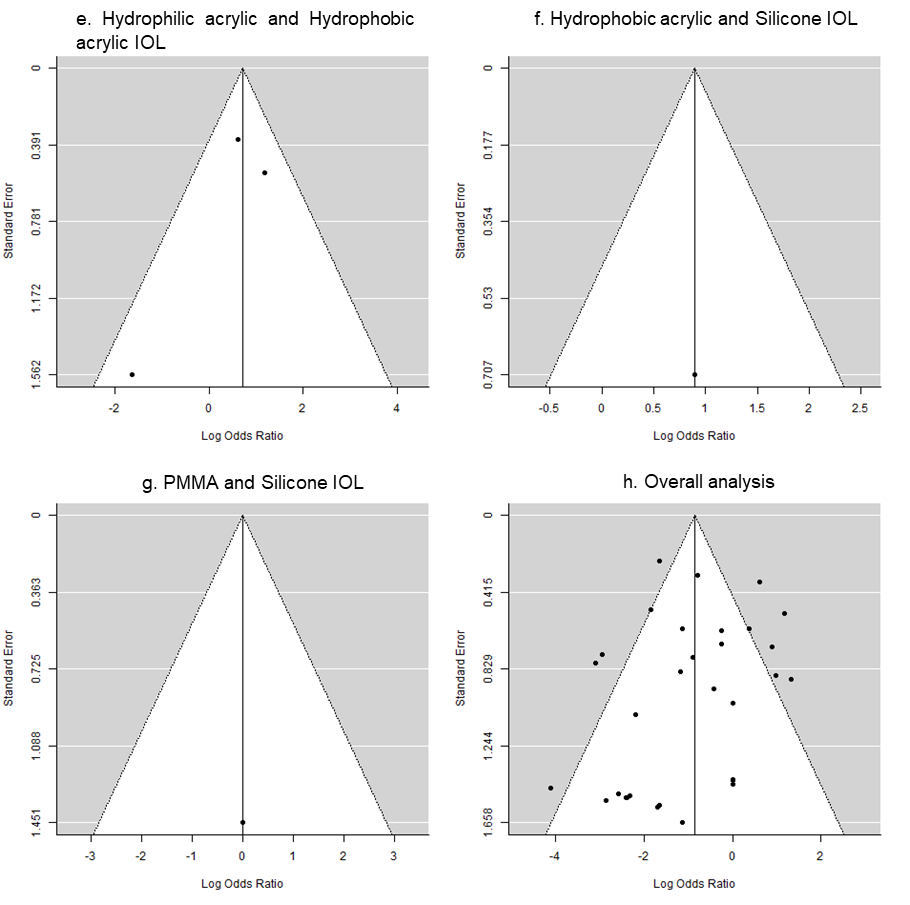

Supplement: S3 File — (ZIP) [file pone.0220498.s003.zip › S3_Files/S3_Fig-2 (e-h).tif]

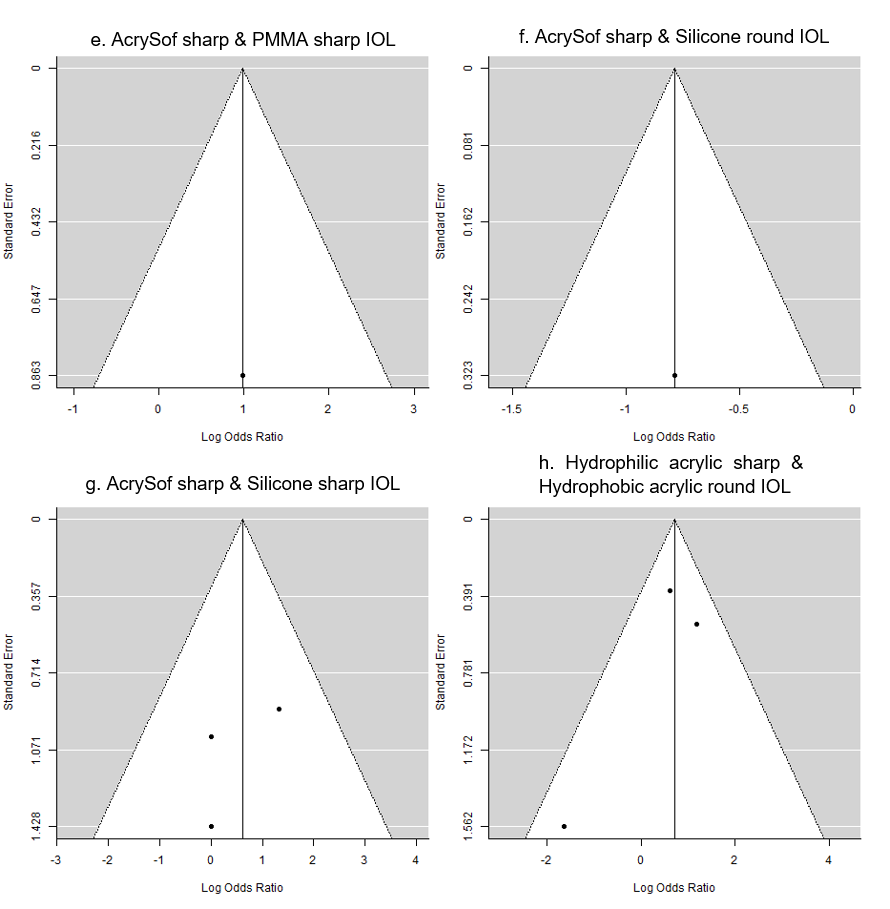

Supplement: S3 File — (ZIP) [file pone.0220498.s003.zip › S3_Files/S3_Fig-3 (e-h).tif]

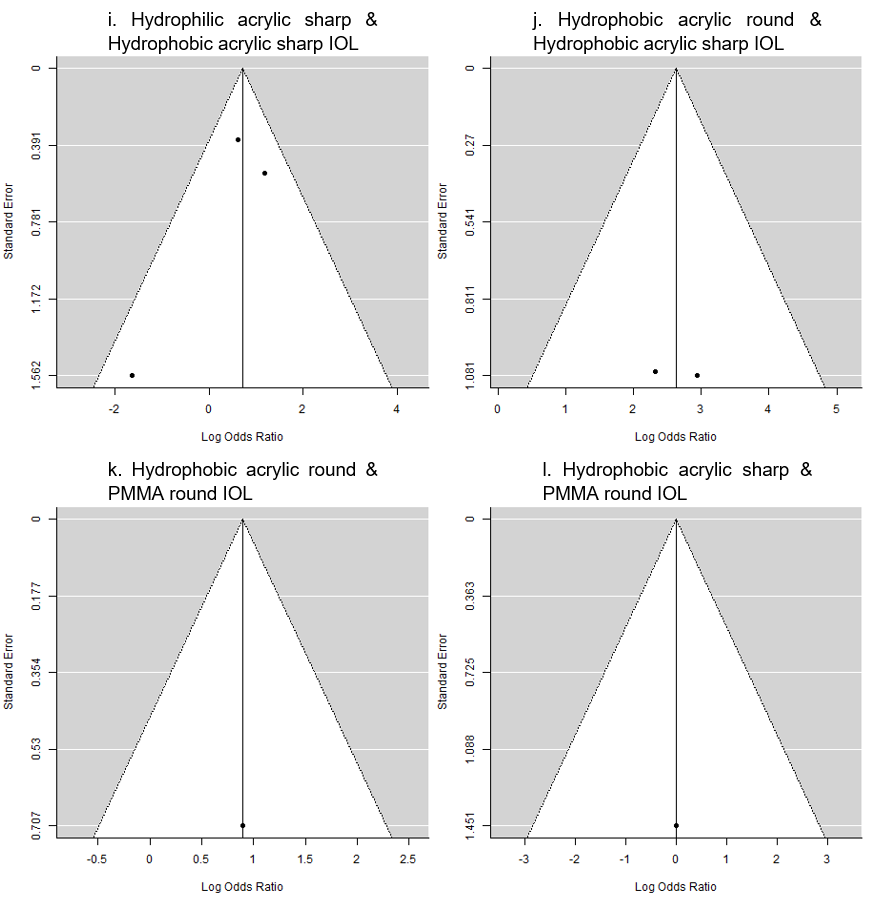

Supplement: S3 File — (ZIP) [file pone.0220498.s003.zip › S3_Files/S3_Fig-3 (i-l).tif]

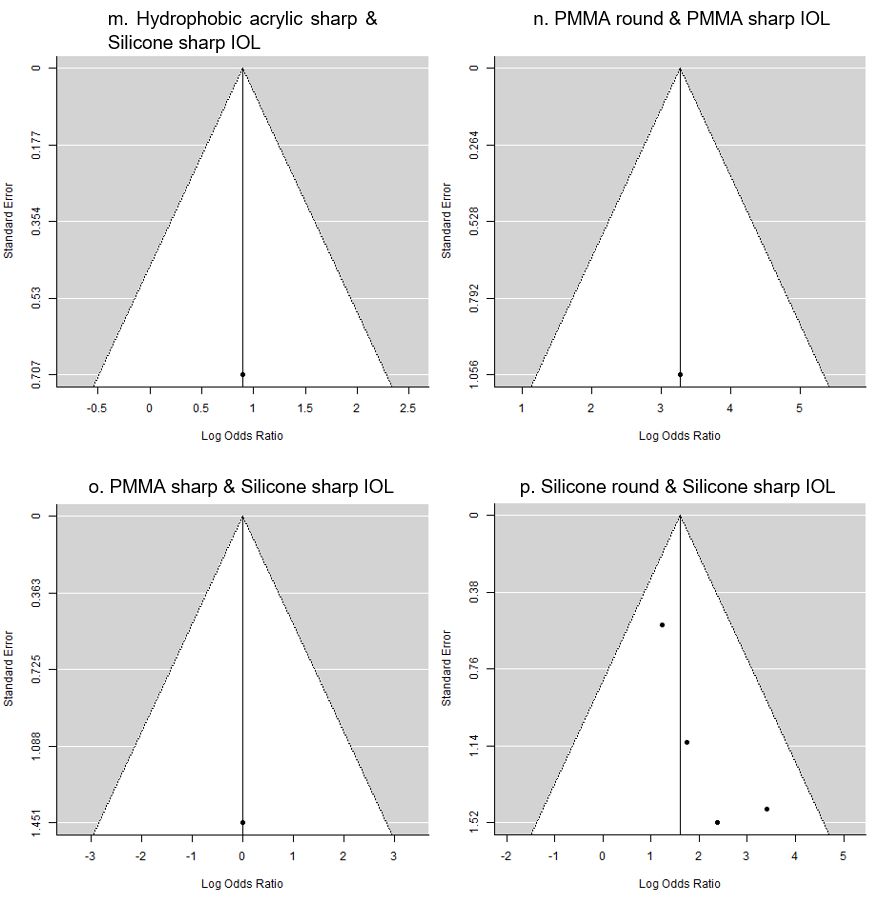

Supplement: S3 File — (ZIP) [file pone.0220498.s003.zip › S3_Files/S3_Fig-3 (m-p).tif]

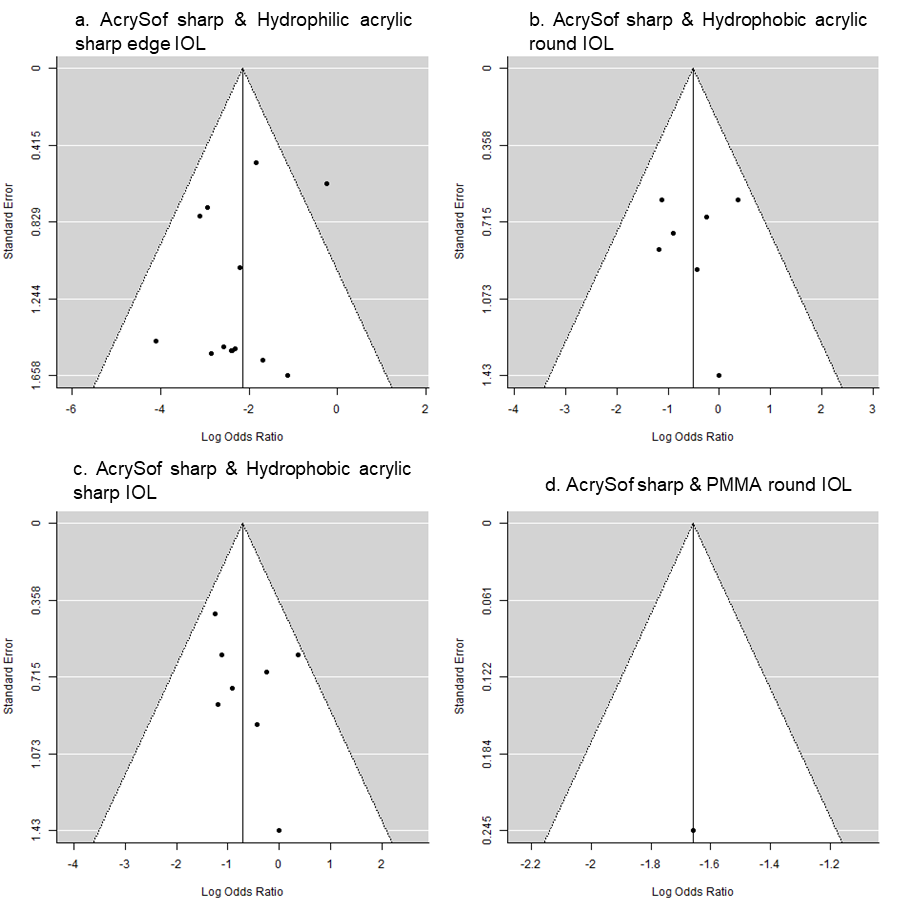

Supplement: S3 File — (ZIP) [file pone.0220498.s003.zip › S3_Files/S3_Fig_3 (a-d).tif]
